# Canvas-Ground Interaction: A New Approach to Quantifying Ground Mechanical Degradation

**DOI:** 10.3390/ma18174041

**Published:** 2025-08-28

**Authors:** Gema Campo-Frances, Santi Ferrer, Diana Cayuela, Enric Carrera-Gallisà

**Affiliations:** 1 Research Group of Conservation of Cultural Heritage, Art and Conservation Department, Fine Arts Faculty, University of Barcelona, 4 Pau Gargallo St, 08028 Barcelona, Spain; 2 Engineer Freelance Researcher, 08173 Sant Cugat del Vallès, Spain; santif1949@gmail.com; 3 Institute of Textile Research and Industrial Cooperation of Terrassa, Universitat Politècnica de Catalunya, 15 Colom St., 08222 Terrassa, Spain; diana.cayuela@upc.edu (D.C.); enric.carrera@upc.edu (E.C.-G.)

**Keywords:** linen canvas, polycotton canvas, ground, mechanical behavior, ground degradation, artificial aging, paintings conservation

## Abstract

Canvases and preparation layers consist of diverse materials that respond differently to mechanical stress. In a canvas painting, elongations and shrinkages can cause deformations—either recoverable or permanent—as well as shear stresses and potential cracks, which may weaken the overall structure. This study aims to better understand the interaction between the canvas and preparatory strata in terms of mechanical behavior. To achieve this, a set of canvases and the same types of canvases with preparation layers were selected. Two types of linen and two types of polycotton were chosen to represent contemporary materials currently available in fine-art stores. Additionally, an accelerated aging process was applied to the samples to compare their mechanical response before and after aging. By examining the mechanical behavior of both primed and unprimed canvases through dynamometric tests, a method to evaluate the mechanical degradation attributable to the ground layer has been developed and explained in detail. This method is applicable to cases with similar characteristics. Analysis of the force/elongation graphs for the ground layer allows for the calculation of how this layer evolves with increasing elongation and how the mechanical degradation worsens. The results highlight the differing mechanical behaviors among the analyzed canvas types in both the warp and weft directions, as well as the degradation values resulting from both the aging process and the dynamometric testing of the canvases and ground layers.

## 1. Introduction

A canvas painting is a collection of heterogeneous materials arranged in different layers that interact with one another. These include the support, canvas, and stretcher, as well as the preparatory, pictorial, and varnish layers. Both the canvas, nailed to its stretcher, and the preparatory layers serve as the support for the pictorial layer. The good condition of the support materials ensures that the pictorial layer, which contains the message of the art work, is well preserved. The materials of the canvases and the preparation layers have very different characteristics and, therefore, exhibit different resistance to elongation. As they interact, the layered structure may deform, resulting in either permanent or recoverable deformation. Breaks or cracks can appear when materials are stressed beyond their elastic limit, weakening the resistance of the painting

Since the 1970s, many researchers have measured the mechanical response of painting materials subjected to elongations up to the end of the elastic phase or until the samples break. Some studies have used uniaxial tests [1,2,3,4] while others have employed both uniaxial and biaxial tests [5,6,7]. Additionally, other researchers have tested unprimed and primed canvas samples in different directions (15°, 30°, 45°, 60°, and 75°) as well as along the warp and weft directions [8,9].

Canvases, due to their structure of perpendicularly intertwined threads (warp and weft), are relatively flexible but very irregular in thickness. Furthermore, differences in textile fibers, yarn thicknesses, spinning systems, and fabric density lead to varied responses to changes in climatic conditions and tensioning. The thickness of the preparation layers applied to the fabric weave is inherently irregular [10]. Additionally, in contemporary preparations made with acrylic resin dispersion, the presence of bubbles can further exacerbate this irregularity [11].

A painting may be displayed or stored in museums with environmentally controlled conditions or outside of such controlled environments where temperature and relative humidity fluctuate over time. In uncontrolled environments, depending on temperature and humidity ranges, the different layers shrink due to the loss of moisture or cooling. Conversely, they swell due to increased moisture or heating. Longitudinal tension in the layers can cause permanent deformations if their elastic limit is exceeded [12,13,14,15,16]. The different longitudinal tensions between layers also produce shear stresses between them. Additionally, the tensioning of the canvas on the stretcher or the action of the wedges can further influence mechanical behavior, potentially exacerbating deformation, stress distribution, and eventual degradation over time.

Between 2022 and 2025, researchers developed new, sophisticated methods to investigate the mechanical properties of the painting materials. In *After the paint has dried a review of testing techniques for studying the mechanical properties of artists’ paint* various recent approaches are reviewed, including models for capturing biaxial stresses and dynamic analyses [17]. To study the interaction between the canvas and the chalk–glue ground, Bury and Bratasz [18] developed a three-dimensional model of a canvas painting, which calculates crack formation sequentially, based on relative humidity variations and ground layer thickness. With a similar approach, a 3D finite elements model to identify crack types and quantify their growth rates under low cycle fatigue has been elaborated [19].

In recent years, finite element modeling (FEM) has been applied to better understand the crack-formation mechanisms, while electronic speckle pattern interferometry (ESPI) has been used to assess fiber resistance and measure strain related to stretcher attachments [16,20,21,22,23,24,25]. In *Understanding Structure, Changing Practice*, Stephen Hackney reviews key conservation advancements from 1955 to the present, discusses their implications for the overall integrity of paintings, and examines the structural and mechanical challenges of canvas paintings. His analysis also refers to the use of ESPI for evaluating tension in canvases mounted on stretchers [26].

To carry out this study, which aims to understand the behavior and interaction between the canvas and the preparatory strata, as well as the mechanisms of mechanical degradation occurring in the ground layer due to stress, a set of four unprimed canvases and their corresponding commercially primed counterparts were selected. Two of the canvases are made of traditional linen, and the other two are contemporary fabrics composed of a mixture of cotton and polyester fibers, commercially known as polycotton. All of these raw canvases and primed canvases are currently sold in fine-art stores. The emphasis of this selection—four types of canvas and four types of grounds—is in representing contemporary canvases and preparations, with a particular focus on having samples of the exact same canvas, both with and without a ground layer. This set of eight samples, tested in both the warp and weft directions, provides the experimental basis for developing a method to quantify mechanical degradation and is not intended as a comprehensive analysis of the mechanical response of all materials.

Linen, made from bast-type vegetable fibers, has historically been the most widely used textile material for canvas and remains the highest-quality fiber for this purpose, which is why many authors have studied its behavior. Numerous studies have also examined other canvas types, such as cotton and polyester fabrics [6]. More recently, research on contemporary canvases made from cotton–synthetic fiber blends have begun to emerge; for example, FTIR analyses have been performed on commercially primed canvases sourced from Australia and Singapore [27,28]. Nonetheless, further investigation is needed to deepen our understanding of these modern canvas materials.

In linen canvases, the weft threads exhibit greater stiffness compared to the warp threads [29,30,31]. The opposite occurs in the polycotton canvases [8,29]. These differences are due to the plain weave structure, where warp threads exhibit greater waviness than weft threads; however, in polycotton canvases, the higher polyester content in the warp threads and the tighter arrangement of the fiber counteracts this phenomenon [4].

The artist canvases that are commercially primed are typically produced as ‘universal’—prepared with a synthetic emulsion—making them suitable for both water and oil-based paints [32]. Canvases sold with a primed coating are typically supplied in rolls at fine-art stores, which requires the ground layers to be thin and flexible.

Since the deterioration of canvases and preparatory layers occurs over time, leading to chemical changes that weaken the material and reduce its mechanical strength, all the samples studied were subjected to an accelerated aging process. Appropriate aging parameters were established for the different materials [33,34,35,36].

## 2. Materials and Methods

To conduct all the tests and observe behavior, a set of four types of canvas and a corresponding set of the same four canvases with commercial preparation layers were selected. All these canvases are currently available in fine-art stores [37,38,39,40]. This set of eight samples, which would be subjected to tensile/elongation tests in both the warp and weft directions, served as a representative case and as the experimental basis for developing a method to quantify mechanical degradation.

The selection of samples was based on the following criteria:

- Comparison of canvas types: the primary criterion was to obtain samples that allow for comparison between the same type of canvas—one without any preparatory layer and one with a commercial preparation layer applied;
- Representation of market varieties: the secondary criterion was to represent a range of canvases currently available on the market, reflecting different qualities used for painting and sold regularly over the years. Therefore, two polycotton canvases and two traditional linen canvases were chosen, to represent both contemporary and classic materials.

**Figure 1:**
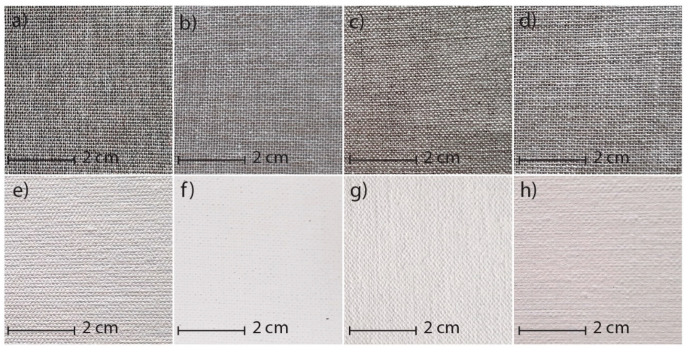
Detail of the selected samples. (**a**) T1 polycotton [37]. (**b**) T2 polycotton [38]. (**c**) T3 linen [39]. (**d**) T4 linen [40]. (**e**) T1P, primed polycotton [37]. (**f**) T2P, primed polycotton [38]. (**g**) T3P, primed linen [39]. (**h**) T4P primed linen [40].

The canvases made from cotton and polyester, commercially known as polycottons, were designated as T1 (T1. Polycotton, Ref: 3210022, Casa Piera, Barcelona, Spain) and T2 (T2. Polycotton, Ref: 0062CR, Barna Art S.L., Barcelona, Spain) The two traditional linen canvases were labelled T3 (T3. Linen, Ref: 066, Claessens Canvas NV/SA, Buggenhout, Belgium) and T4. (T4. Linen, Ref.: T300S; Vicenç Piera, Barcelona, Spain). The same canvases with applied preparation layers are referred to as T1P (T1P. Primed Polycotton, Ref: 3210026, Casa Piera, Barcelona, Spain), T2P (T2P. Primed Polycotton, Ref: 0062CR, Barna Art S.L., Barcelona, Spain), T3P (T3P. Primed Linen, Ref: B166, Claessens Canvas NV/SA, Buggenhout, Belgium), and T4P (T4P. Primed Linen, Ref.: T300S-P, Vicenç Piera, Barcelona, Spain). (See Figure 1).

### 2.1. Textile Identification and Characterization

To confirm that the canvases with and without a ground layer are identical, all samples were examined under a stereoscopic microscope Olympus SZX7 (OLYMPUS CORPORATION, Tokyo, Japan). This examination also revealed specific characteristics of the preparatory layers and their application to the canvas, which will be detailed later.

By preparing the fibers and examining them using optical Olympus BX51 (OLYMPUS CORPORATION, Tokyo, Japan) and reagents, a qualitative identification of the textile fibers in the four types of canvases was performed [41,42,43]. For identifying the synthetic fibers in the polycotton canvases, analytical methods involving solvents and heating were employed [44,45].

A quantitative identification was also performed according to the relevant standard [46] to determine the exact proportion of each fiber in the blended polycotton canvases. For polycottons, which exhibit differences in appearance and composition between the warp and weft threads, qualitative identification was conducted separately for each, after removing compounds such as sizing and finishing as prescribed by the corresponding ISO [46]. Specific procedures for analyzing yarns with cotton blends and yarns with viscose blends were applied [47,48].

A detailed analysis of the yarns, including linear density, spinning type, and yarn sizing materials, as well as an examination of the fabrics, including weight per square meter, the number of warp and weft threads per linear centimeter, and the nominal thickness of the canvas, can be found in a previous study where these characteristics were determined [4].

### 2.2. Ground Identification and Characterization

As noted, all the grounds were commercially prepared. Their characterization involved observations using a stereoscopic microscope and optical microscopy with polarized, incident, and transmitted light, under both halogen and UV illumination to examine cross sections. This allowed for a detailed examination of the layer arrangement and any irregularities in their distribution.

The identification of pigments and fillers in the ground layers was carried out using the following techniques and equipment: optical microscopy (Olympus BX54, (OLYMPUS CORPORATION, Tokyo, Japan) with polarized, incident and transmitted light under halogen, and UV illumination with an attached Olympus DP74 camera.

Scanning electron microscopy with energy-dispersive X-ray spectroscopy (SEM-EDX) was conducted with the Quanta 200 de FEI instrument (FEI Company, Barcelona, Spain) operated by low vacuum-microanalysis Oxford Instruments. X-ray microdiffraction (M-DRX) was performed with a Bruker D8 Discover A25. (BRUKER Company, Madrid, Spain). The binder materials in the preparation layers were identified using gas chromatography–mass spectrometry (GC-MS) with an Agilent Technologies 6890N GC–5973 MS system (AGILENT TECHNOLOGIES, Wilmington, DE 19808-1610 EE. UU.), and with a Fourier transform infrared spectroscopy (FTIR) with a PerkinElmer Spectrum Two instrument (PERKINELMER SCIENTIFIC SPAIN SL Madrid, Spain).

### 2.3. Aging Procedure

To determine the temperature and relative humidity (RH) conditions for aging the samples, three key factors were considered:

- Polyester fibers in canvas samples:

Among the canvas samples, four (T1 and T2 in both warp and weft directions) contained polyester fibers in proportions ranging from 29 to 66%. According to the *Polymer Data Handbook* [49], the glass transition temperature (Tg) of polyester fibers ranges between 70 and 115 °C;

- Linen canvas samples:

Four of the canvas samples (T3 and T4 in both warp and weft directions) were made of linen. According to Zervos [34], the aging process for cellulose—the main component of linen—is relatively uniform within the range of 60 °C to 90 °C and 30% to 80% relative humidity. Moreover, a temperature increase of only 5 K can double the degradation rate;

- Primed samples with ground layers:

All the primed samples had ground layers adhered to the canvases. These ground layers contain acrylic resins mixed with fillers such as calcite, dolomite, titanium white (rutile), and talc. According to Feller [50], cross-linking in acrylic resins occurs exclusively at temperatures significantly above the Tg. However, at temperatures slightly below the Tg, varying degrees of chain scission can occur. This leads to an observed transition from chain scission to cross-linking.

Above the Tg, polymer molecules—particularly the ester side chain groups—exhibit significant thermal motion. This increased molecular movement allows free radical sites (unshared electrons) within the polymer structure to interact and link with neighboring chains, facilitating cross-linking. In contrast, at temperatures below the Tg, molecular motion is restricted or “frozen”, preventing the formation of cross-links between adjacent chains.

The Tg of acrylic resins varies between 25 °C and 100 °C, depending on the specific resin type [51]. In some acrylic resins, the Tg is higher before aging, while in others, it increases after aging [52].

Considering the above factors, a temperature of 78 °C and 75% relative humidity (RH) was applied for 800 h in a Climatic Test Chamber Angelantoni ACS KEYKRATOS PLUS (Angelantoni Test Technologies, Massa Martana, Italy).

These conditions are between the ones in the ISO-5630 part 1 (Dry treatment at 105 °C) [53] and part 3 (80 °C and 65% RH) [54] and allow the samples to age enough, limiting the vapor absorption in the linen and cotton fibers. The selected aging temperature of 78 °C, in some cases, was higher than the Tg of the ground acrylic resins in the primed samples; fillers such as calcite and others can lead to cracking, a phenomenon not observed in studies of unfilled acrylic resins.

Selecting aging parameters for canvas–ground systems is challenging due to the varied stability of their components. Our aim was not to simulate museum conditions but to apply a controlled accelerated-aging cycle (78 °C, 75% RH, 800 h) to compare mechanical performance before and after aging. This protocol balances cellulose and polyester degradation while avoiding excessive damage to acrylic resins.

Before placing the materials in the climatic chamber, the canvases, primed and unprimed, were nailed to a stretcher to simulate the tension conditions of a painting canvas along the aging process. The samples were then cut to perform the mechanical resistance (dynamometric) tests, avoiding the areas of the canvas in contact with the stretchers. The stretchers were placed in the chamber with metal spacers between them to facilitate airflow.

### 2.4. Mechanical Tests

All analyses and textile characterization tests were carried out under controlled room temperature and humidity conditions (20 °C ± 2 and 65% ± 4 RH), as per ISO 139:2005 standards [55]. This ensured consistent conditions for the reliable comparison of results across different materials.

**Figure 2:**
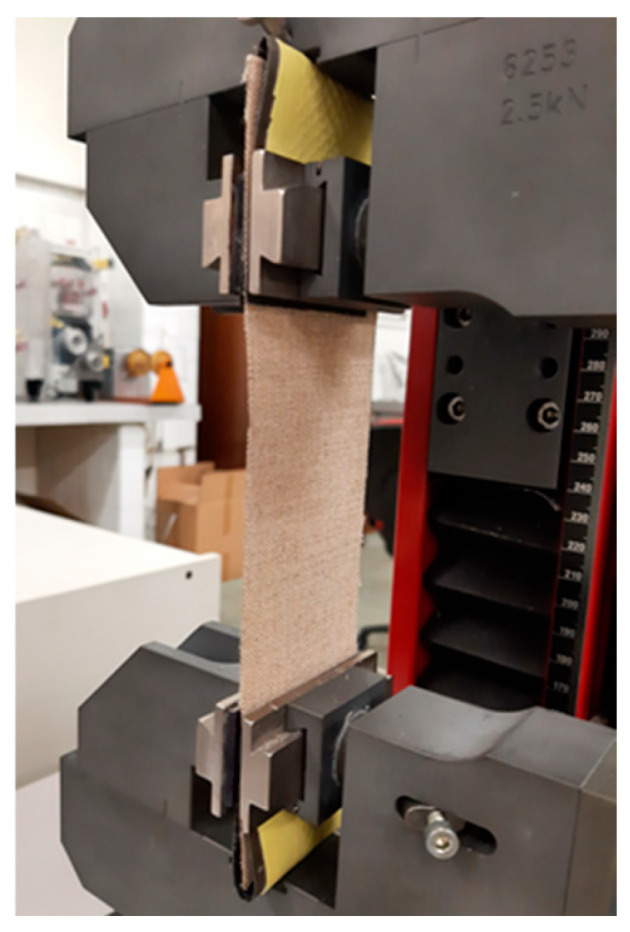
Detail of the clamping system used during tension/elongation tests to prevent sample slippage.

The mechanical resistance to the forced elongation of the samples was measured by dynamometric tests, before and after the aging process. To prevent sample slippage, a double layer of sandpaper and rubber film was applied between the clamps and the sample (see Figure 2). There were 32 analyzed cases of new and aged canvases of T1, T2, T3, T4, primed and unprimed, for both warp and weft directions. A Zellweger Uster TENSOKID (SRI KARPAGAPRIYA TEXTILES PRIVATE LTD. Rajapalayam, India) equipped with a 2500 N static load cell was used. Rectangular samples 200 mm × 53 mm between clamps were cut parallel to the warp or the weft direction. The measurements were carried out at a constant extension rate of 60 mm/min, sufficiently accurate for these tests, up to the breaking point. The force was measured in Newtons as a function of elongation measured in %. For each case, 5 samples were tested and the average values were calculated.

### 2.5. Method for Calculating the Degradation Attributable to the Ground Layer in Polycotton- and Linen-Primed Canvases

The mechanical behavior of a canvas indicates that before reaching the elastic phase, the canvas progresses through its first phase (slack and bedding-down zone) and its viscoelastic second phase, which corresponds to crimp removal [16].

By applying the methods developed by Young [29], Young and Hibberd [7], and Dowling [56] for linen, polyester, and cotton/polyester canvases, for linen, one can simulate the canvas’s behavior when the force decreases at any given elongation. The slope of the initial decreasing force/elongation curve is slightly less than that of the elastic phase and decreases further as the force approaches zero. When the force increases in the next cycle, the slope rises and remains nearly linear up to the original force and elongation. With repeated cycles, the hysteresis diminishes, and the slopes during both the descent and ascent match those of the elastic phase.

By subtracting the force measured in the unprimed sample from that in the primed sample at the same elongation, we can determine the force corresponding to the ground layer at that elongation. Using these values, we obtain the ground force and elongation curve from zero up to the point of sample break.

The elastic phase of the ground layer occurs at elongations where the canvas is still in its second, viscoelastic phase. The interaction between the ground and the canvas, after the ground’s elastic phase, can lead to the ground degradation with varying degrees of permanent deformation. In some cases, cracks may appear.

The ground layer applied to the canvas has a very variable thickness. Consequently, shear forces between the canvas and the ground—and the stresses in each—vary by location and direction. According to the ground thickness and the interactions with the canvas geometry along the warp and weft directions, the resistance to the applied forces also varies along the ground layer.

Two methods have been developed to quantify ground degradation:

1. Total ground degradation compares the energy absorbed by the ground layer during the second cycle versus the one in the first cycle, both up to the end of the elastic phase in the primed samples. The resulting degradation is expressed as a percentage of energy loss (*Ground Degradation%*). Since energy is measured in *Joules* (*N·m*), and all samples have the same length and width, we compare the energy values using the energy area ratio in (*N%*) instead of (*N·m*).

To calculate the total degradation attributable to the ground layer, in both new and aged samples, during the first and second cycle we use the *P*, *Q*, and *R* values.

First, we summed all the trapezoidal areas ∑ [(*F_j_* + *F*_*j*−1_)/2] · ∆*ε_j_* from zero in the ground force/elongation first cycle. For this calculation, we used the *F_j_* and *ε_j_*, values provided in in the dynamometric tests.

In linen the trapezoidal areas extend up to the end of the 3rd phase and in polycotton they extend up to the 4th phase.

To calculate *P new*, we used Equation (1) with the *F_j_* and *ε_j_* values from the new samples

*P new* = ∑[*ε _i_ (%)* − *ε*_*i*−1_ (%)] · [*F*_*i*−1_
*(N)* + [*F_i_ (N)* − *F*_*i*−1_
*(N)*]/2] for *i* from 1 to *n* (1)

and for *P aged* we used the same equation, but with the *F_j_* and · *ε _j_* values from the aged samples.

*Q* is the area of the triangle, measured in *(N%)*, corresponding to *F ·* ∆*ε*/2 for the ground during the 2nd cycle of the elastic phase, where *F* is the force *(N)* at the end of the 2nd cycle and ∆*ε* is the elongation *(%)* increase during that cycle. *R* is the area calculated as *P* − *Q*, also expressed in *(N%)* units. Both *Q* and *R* were calculated for the new and aged samples (*Q new*, *Q aged*, *R new*, *R aged*), with Equations (2) and (3).

*Q* = [*Ground F 3rd phase (elastic) (N)* · ∆*ε* 2*nd cycle* (%)]/2 (2)

(3) $$\begin{array}{c} P=Q+R=\sum [{\varepsilon \;}_{I}\;(\%)-\varepsilon_{i-1}\;(\%)]\;\cdot \;[\mathrm{Ground}\;F_{i-1}\;(N)+[\mathrm{Ground}\;F_{i}\;(N)-\mathrm{Ground}\;F_{i-1}\;(N)]/2]\\ \mathrm{for}\;i\;\mathrm{from}1\mathrm{to}n. \end{array}$$

Using these data (*P*, *Q*, and *R*), the total ground efficiency (*Ground Eff%*) and the total ground degradation (*Ground Degr%*) can be calculated for the new and for the aged samples with the same Equations (4) and (5) but with the different *Q* and *P* values for new or aged samples.

*Ground Eff%* = 100 · [*Q/P*] = 100 · [*Q/(Q + R)*] (4)

*Ground Degr%* = 100 · [1 − *(Q/P)*] = 100 · [1 − *(Q/(Q + R)*] (5)

The ∆% *Aging* variable corresponds to the difference between the *Ground Degr%* of the new and aged samples, quantifying the degradation in the ground layer due to the aging process.

It is not possible to calculate the *Ground Degr%* for linen primed samples in the weft direction, as the force–elongation graphs of the primed and unprimed samples intersect or overlap;

2. Bedding-down degradation assesses degradation at the end of the first phase, capturing the ‘*bedding-down*’ effect. The ground degradation at the bedding down (*Ground bed Degr%*) can be calculated as

*Primed canvas bed (N · %)* = *F (N)* · [*ε bed end (%)* − *ε bed start (%)*] (6)

*Unprimed canvas bed (N* · *%)* = *F (N)* · [*ε bed end (%)* − *ε bed start (%)*] (7)

*Ground bed (N* · *%)* = *Primed canvas bed (N* · *%)* − *Unprimed canvas bed (N* · *%)* (8)

*If (Ground bed (N* · *%)* < 0 *or Ground bed (N* · *%)* = 0*)*
*then Ground bed Degr (%)* = 0

*else Ground bed Degr (%)* = 100 · [1 − *((Ground bed (N* · *%))/((Q* + *R) N* · *%))*] (9)

## 3. Results and Discussion

### 3.1. Textile Identification and Characterization

Canvases T1 and T2 have different compositions in their warp and weft threads and contain varying mixtures of cotton, polyester, and viscose fibers, as detailed in Table 1. In these two polycotton canvases the proportion of cotton in the weft threads surpasses that of the warp, reaching a maximum of 71% in T2. T1 has the highest polyester content, averaging 50% across both warp (66%) and weft (33.5%). It is also the only canvas that contains viscose, averaging 25% in both warp and weft. The presence of polyester in the warp yarns of T1 enhances their mechanical resistance, as demonstrated by the results of the mechanical tests. In the context of potential restoration procedures involving heat on canvases with a high polyester content, precautions must be taken to prevent fiber fusion, which may occur above the glass transition temperature (Tg) [57].

In contrast, canvases T3 and T4 are composed entirely of linen, with 100% flax fibers identified in both the warp and weft yarns.

Table 1 summarizes the fiber identification for all canvases, including their weight per square meter, thread count per centimeter, and the spinning system of their yarns.

The yarns of the polycotton canvases T1 and T2 are produced using an open-end spinning system, a modern and cost-effective method that enables greater production efficiency. The warp yarns feature tightly packed fibers, resulting in more compact threads, while the weft yarns are thicker and exhibit a less dense, more disordered fiber structure. These substantial differences between the warp and weft threads in polycottons are likely to affect their mechanical response and the types of breaks that may occur in the canvas [4].

The two linen samples, T3 and T4, have a traditional ring spinning yarn with a smooth Z twist in warp and weft yarns.

### 3.2. Ground Characterization and Identification

The organization of the ground layers for all samples, as well as the identification of the pigments and fillers within them, can be found in Table 2. In the case of T1P, three layers can be distinguished, while T2P has two layers (see the large white arrows in Table 2 indicating the thickness of the weft threads, while the small arrow points to a fine substratum within the ground layer). Both T3P and T4P have a single preparatory layer, although T3P also exhibits a thin layer of organic material applied as a sizing (see the small white arrow pointing to it in Table 2). In the same table, the white arrows in T4P indicate the thickness of both the weft and warp threads in the canvas. These results of the samples’ stratigraphy are consistent with those analyzed in other studies of contemporary commercially primed canvases [27].

**Figure 3:**
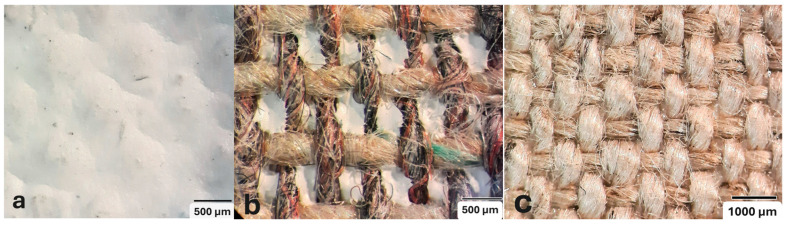
Surface and ground transfer characteristics of the samples: (**a**) Irregularities on the front surface of T3P; (**b**) Very high ground transfer on the reverse side of T1P; (**c**) No ground transfer observed on the reverse side of T3P.

Regarding the thickness of each of these layers and sub-layers, it varies between 25 and 75 microns, as detailed in Table 2. It is important to consider that the thickness of a preparatory layer applied to a canvas—an inherently uneven surface due to the interweaving of warp and weft threads—will naturally be irregular. Images from the stereoscopic microscope illustrate how the preparatory layers adapt to the fabric’s texture and the warp–weft structure (see Figure 3a). Likewise, the transfer of preparation material to the reverse side varies significantly between samples, depending on the openness or density of the canvas structure (see Figure 3b,c).

**Figure 4:**
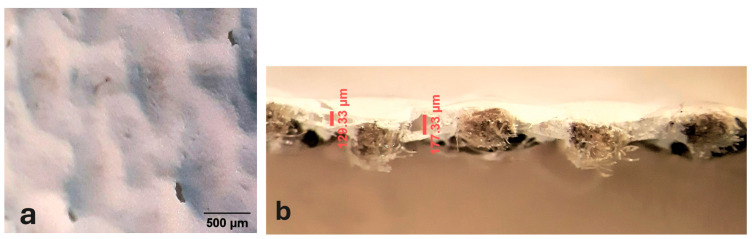
Holes in T1P: on the surface (**a**) and in the cross section (**b**).

Some of the preparatory strata exhibit distribution defects, either on the surface or within their internal structure. These defects primarily manifest as holes that disrupt the continuity of the preparation layer, particularly in sample T1P, where they are visible in the most recessed areas of the thread interlacing (see Figure 4a,b).

**Figure 5:**
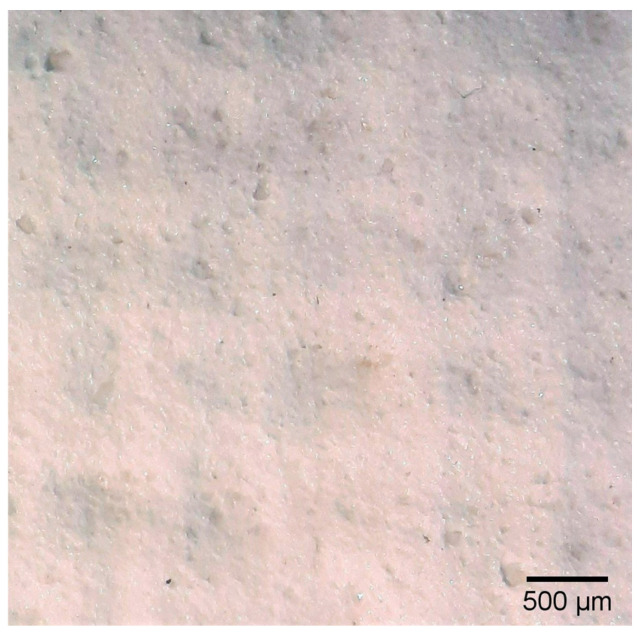
Close-up of the ground surface of the T4P sample, where solid filler particles are visible.

The results of the identification of pigments and fillers indicate that the most prevalent pigment is titanium white, combined with various forms of calcium carbonate, such as calcite and dolomite, which are widely used as fillers (see Table 2). In sample T4P, talc, a silicate commonly used as a filler, was identified, along with other fillers such as calcite, dolomite, and the pigment titanium white. Some of the solid particles from these filler materials can be observed on the surface of the T4P sample ground, with sizes of up to 150 microns (see Figure 5).

Barium sulphate, present in sample T2P, appears as the mineral barite, the main commercial source of barium, valued for its opaque white appearance and high density. Refer to Table 3 for the X-ray microdiffraction analysis results, detailing the pigments and filler materials found in the ground layers of each sample. These identified pigments and fillers align with those commonly used in contemporary grounds [11].

Regarding the binders of the grounds, acrylic resins were identified in all four samples. Many commercially available preparations today contain a water-based acrylic dispersion resin binder composed of either an ethyl acrylate/methyl methacrylate (EA/MMA) or butyl acrylate/methyl methacrylate (BA/MMA) copolymer resin as described in [11] and [28]. Combinations of these various synthetic resins, accompanied with stabilizers and emulsifiers, are commonly found in primed canvases currently available on the market [27].

### 3.3. Dynamometric Tests and Phases Analysis

To discuss and analyze the results of the dynamometric tests, we refer to the four phases of the force/elongation process. These phases help explain the different phenomena that occur in the canvas and ground layers as the applied force increases.

The **first phase**, between approximately 0 *N* and 20 *N*, is characterized by elongation that is nearly proportional to the applied force. This phase includes the slack zone and the bedding down, where elongation occurs with a minimal increase in force.

The **second phase** involves viscoelastic behavior in both primed and unprimed canvas; however, the ground layer does not undergo this phase. In the canvas, this phase is characterized by crimp removal and fiber adjustment, which occur in both types of samples—primed and unprimed. Crimp removal refers to the straightening of the natural waviness or bends (crimps) in the yarns that make up the canvas fabric. In this viscoelastic phase the increase in force rises with increasing elongation. As the crimps are progressively removed, the fibers align more closely with the direction of the force. During this phase, elongation is no longer proportional to the applied force, indicating a shift from elastic to viscoelastic behavior.

The **third phase** in both the canvas and the ground corresponds to their elastic behavior, but it occurs at significantly different elongations. The ground enters its elastic phase at very short elongations, while the canvas reaches this phase only after much greater elongation. Once the ground exits its elastic phase, its Young’s modulus decreases, indicating a loss of stiffness and the onset of permanent deformation. Permanent deformation refers to a change in shape or structure that remains even after the applied force is removed. In this state, the ground material does not return to its original dimensions, meaning the deformation is irreversible. In contrast, the canvas remains in its viscoelastic phase for a longer period before transitioning into its own elastic third phase.

During the **fourth phase**, both the canvas and the ground undergo irreversible deformation, and by the end of this phase, material failure occurs, resulting in the fracture of the sample. In this phase, the materials often exhibit unstable and erratic mechanical responses, characterized by sudden fluctuations in force and deformation. This behavior is more common on the canvas than on the grounds. In the canvas, cracking often initiates along fiber bundles or at points of stress concentration, while in the ground layer, cracks can form due to brittleness and reduced elasticity. See a summary of the behavior of the samples during the dynamometric phases in Table 4.

From this point forward, tables and figures whose names start with ’S’ refer to those in the Supplementary Materials.

**Figure 6:**
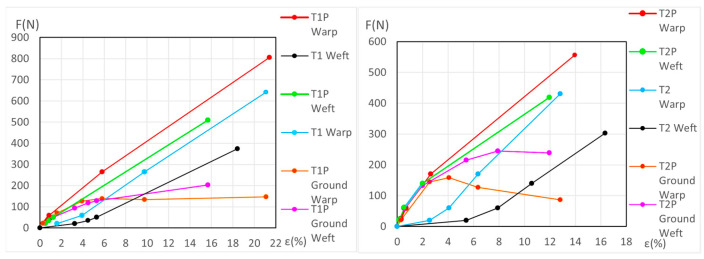
Force–elongation graphs of the new polycotton samples T1-T1P (**left**) and T2-T2P (**right**) both for warp and weft directions up to the end of the 3rd phase.

**Figure 7:**
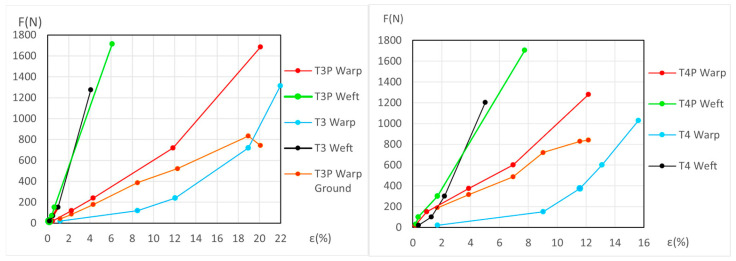
Force–elongation graphs of the new linen samples T3-T3P (**left**) and T4-T4P (**right**), both for warp and weft directions up to the end of the 3rd phase.

**Figure 8:**
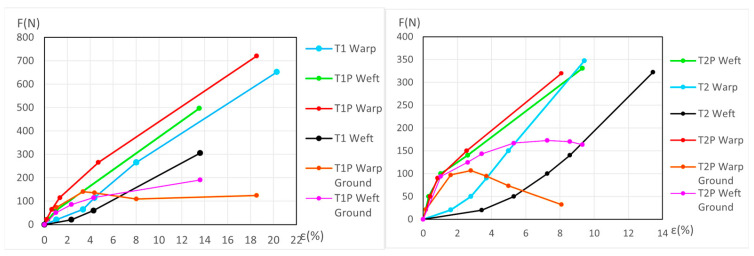
Force–elongation graphs of the aged polycotton samples: T1-T1P (**left**) and T2-T2P (**right**), both for warp and weft directions up to the end of the 3rd phase.

**Figure 9:**
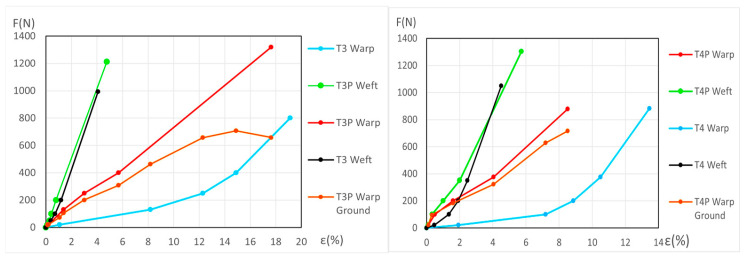
Force–elongation graphs of the aged linen samples: T3-T3P (**left**) and T4-T4P (**right**), both for warp and weft directions up to the end of the 3rd phase.

The force (*F*) and elongation (*ε*) results for the new samples in both the warp and weft directions across the 1st, 2nd, and 3rd phases are shown in Figure 6 (polycotton T1-T1P and T2-T2P) and Figure 7 (linen T3-T3P and T4-T4P) and are summarized in Table 5. For the aged samples, refer to Figure 8 and Figure 9 and Table 6. Table 5 and Table 6 also include the maximum force in the fourth phase, as well as the force and elongation where the sample breaks. The transitions between the first, second, and third phases are marked with a dot (—●—). In the viscoelastic second phase, additional points are included to denote sub-phases, enhancing precision. The force and elongation values for these sub-phases in new samples are detailed in Table S1 for new samples and in Table S2 for aged samples.

In the legend of the graphs, the term “Ground” refers to the ground material of the primed samples.

Before the end of the first phase, the elongation increases without a corresponding increase in force. This phenomenon, known as bedding down, occurs in both primed and unprimed samples. Although not visible on the scale of Figure 6, Figure 7, Figure 8 and Figure 9, the corresponding *F/ε* and *ε/εbed* ratios are provided in Table 4 and Table 5. As a representative example, bedding-down graphs for the new T2 warp and T2P warp samples are shown in Figure S1. Bedding down was observed in all the tested samples, with values ranging from 343 *N/m* in the aged T3P weft samples to 547 *N/m* in the new T4P weft samples. The ground degradation caused by bedding-down degradation was also calculated and found to be very small (see Tables S22 and S23).

For primed and unprimed, new and aged samples, according to the forces/elongation values obtained in the dynamometric tests, we defined the subphases along the second viscoelastic phase, which is not lineal. The third elastic phase and each second subphase are associated at a lineal graph and this produces a polygonal with two to four segments adjusted at the graph. Each segment is associated at the (10) lineal equation with the *A _j_* and *B j* coefficients corresponding to each second subphase and the third phase. These coefficients are provided in Tables S3–S6.

*F_j_ = A _j_ · ε _j_ + B _j_* (10)

Table S7 provides an example of the calculation method, used to obtain the *A _j_* and *B _j_* coefficients for the new T1 warp sample in the second A subphase, the second B subphase, and the third elastic phase.

Using Equation (10) and the values of *ε*, *A _j_*, and *B j* for the unprimed samples in each case, direction, and phase or subphase, the force at the same elongation for the corresponding primed sample was calculated. The same procedure was then followed using the *ε*, *A _j_*, and *B j* values for the primed samples, calculating the force at the same elongation for the corresponding unprimed sample. The difference between the force of the primed sample and the unprimed sample at the same elongation corresponds to the force applied to the ground at each of these elongations. The ground graphs obtained are polygonals and the segments are between five and eight segments. These values of *F* and *ε* for the ground are displayed in Figure 6, Figure 7, Figure 8 and Figure 9 but cannot be used for T3P weft and T4P weft, both new and aged.

Figure 7 shows that the force/elongation graphs for the new T3P weft and T4P weft samples intersect with those of T3 and T4, respectively. In Figure 9, this intersection occurs only in the aged T4P weft, while the aged T3P runs parallel very close to T3. This fact, the intersection and it running in parallel very closely, does not allow a correct calculation of the force/elongation graphs for the ground layer, nor the mechanical ground degradation in these linen weft samples.

Based on the evolution of the force-to-elongation relationship throughout the elongation and contraction cycles, we developed a method to quantify the permanent mechanical degradation of the ground layer. This method was applied to the polycotton-primed samples, in both warp and weft directions, and to linen-primed samples in the warp direction, all up to the end of the third phase.

#### 3.3.1. General Evolution of the Samples 

**Figure 10:**
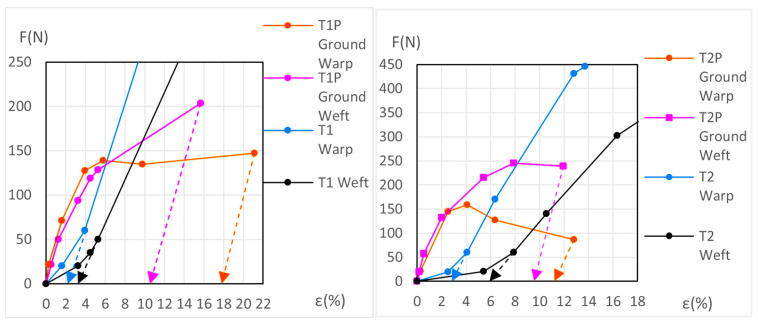
Detailed view of new T1P ground and T1 canvas (**left**) and new T2P ground and T2 canvas (**right**).

**Figure 11:**
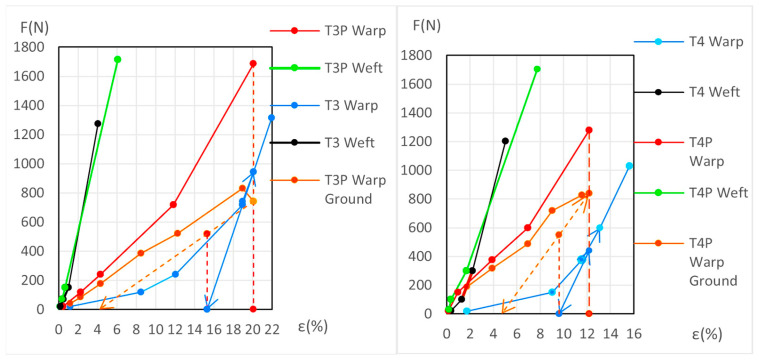
Detailed view of new T3-T3P warp (**left**) and new T4-T4P warp (**right**) up to the end of the 3rd phase. Ground and canvas warp with elastic decreasing to F = 0.

**Figure 12:**
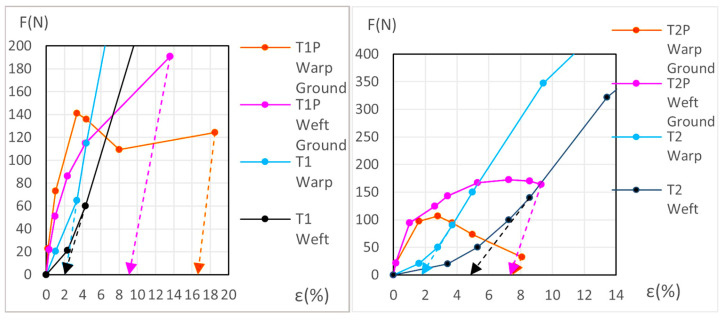
Detailed view of aged T1P ground and T1 canvas (**left**) and aged T2P ground and T2 canvas (**right**).

**Figure 13:**
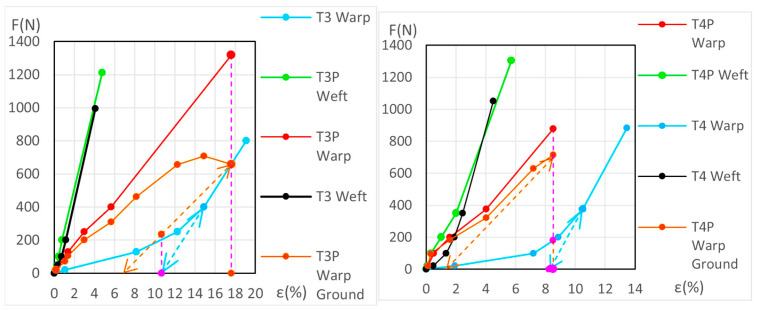
Aged T3-T3P (**left**) and aged T4-T4P (**right**) up to the end of the 3rd phase. In both ground and canvas warp with elastic decreasing to *F* = 0.

The dynamometric evolution of the new samples is shown in Figure 10 for poly-cotton and in Figure 11 for linen. For the aged samples, the results are shown in Figure 12 for polycotton and in Figure 13 for linen.

In the polycotton-primed samples, the ground’s elastic phase occurs at very short elongations, while the canvas reaches its elastic phase at much longer elongations. If the sample is allowed to shrink, along or near the ground’s elastic phase, the ground shrinks more than the canvas and therefore the canvas wrinkles. At greater elongations, when the ground is in the fourth phase and the polycotton canvas is still in its elastic phase, increasing the applied force results in a greater elongation of the ground, although the corresponding force increase is minimal (the curve has a very small slope or even descends). The applied force on the primed sample can then be reduced until the ground force reaches zero. At that point, both the canvas and ground share the same elongation, but the canvas still supports a positive force. If the force continues to decrease to zero, the canvas elongation reduces according to its elastic behavior. During this process, the ground contracts, wrinkles, and undergoes plastic deformation. Meanwhile, the polycotton canvas shrinks along its elastic phase, without undergoing plastic deformation; its change is due only to fiber adjustment during the viscoelastic phase. 

In the primed warp linen samples, behavior opposite to that observed in the poly-cottons samples appears at higher elongations. During the elastic phase of the canvas, the ground layer sustains a higher force than the canvas. If the elongation of the primed sample is reduced, the canvas follows its elastic phase down to zero force, decreasing its elongation accordingly, while the ground still maintains a positive force at that elongation. If the ground force is reduced to zero, the ground elongation also shortens. As a result, the canvas contracts and wrinkles.

During the elastic phase, in the weft primed and unprimed linen samples are very close and mostly intersect. This intersection, or the very close position, prevents the accurate quantification of the ground layer’s degradation progression during the dynamometric tests for these samples. It happens in the new and aged weft linen samples.

#### 3.3.2. Detailed Evolution of New Polycotton Samples T1P and T2P

At the end of the third (elastic) phase, the T1 warp (canvas) reaches a force of 642 *N* at an elongation of 21.1%. At this same phase endpoint, the T1P warp reaches 806 *N* at 21.42% elongation. At 21.1% elongation, the ground layer carries a force of 147.2 *N*. If the T1P warp begins to contract, the ground also contracts, following the slope of its elastic phase, from 71.3 *N* to 20 *N*, and attains the force 0 *N* at an elongation of ε = 17.8%. At this point (ε = 17.8%), the canvas is still under tension, while the ground is not. If the T1P warp continues to shrink, the canvas follows the same *F/ε* slope as the T1 warp in its elastic phase, and the force reaches 0 *N* at an elongation of ε = 2.05%. Between *ε* = 17.8% and *ε* = 2.05%, the ground continues to contract and wrinkle, resulting in plastic deformation. See Figure 6 (left) and Figure 10 (left).

For the T1P weft new samples, see Figure 10 (left) and the explanation in Supplementary Materials, Figure S2 (Detailed evolution of new samples T1P Weft (Polycotton)).

For the T2P new samples, both in the warp and weft directions, see Figure 10 (right) and the explanation in Supplementary Materials, Figure S3 (Detailed evolution of new samples T2P Warp and Weft (Polycotton)).

#### 3.3.3. Detailed Evolution of New Samples T3P Warp (Linen)

If the tension in the T3 warp (canvas) is reduced from the beginning of its elastic phase (ε = 18.96% and 742 N) to 0 N; it contracts to 15.3%, following the slope of its elastic phase. At the end of its elastic phase, the T3P warp reaches 1686 N at 20.1% elongation. If the T3P warp reduces its elongation to 15.3%, the canvas force drops to 0 N while the ground retains a force of 518.38 N. If the ground then continues to release tension until it also reaches 0 N, the T3P warp follows the slope of the ground’s elastic phase, and the elongation decreases further to ε = 4.17%. The canvas—initially at 15.3% and 0 N—contracts and wrinkles, resulting in permanent deformation. See Figure 11 (left).

#### 3.3.4. Detailed Evolution of New Samples T4P Warp (Linen)

See Figure 11 (right), and the explanation for the new T4P warp samples, in Supplementary Materials, Figure S4 (Detailed evolution of new samples T4P Warp (Linen). This process is similar to the new T3P warp (Linen).

#### 3.3.5. Detailed Evolution of New Linen Samples T3P and T4P Weft

During the elastic phase shown in Figure 11 (left), the curves representing the new T3P weft (primed) and T3 weft (unprimed) are very close and intersect. A similar overlap occurs in Figure 11 (right) between the new T4P weft (primed) and T4 weft (unprimed). This intersection prevents accurate quantification of the ground layer’s degradation progression during the dynamometric tests for these samples.

#### 3.3.6. Detailed Evolution of Aged Samples T1P and T2P (Primed Polycotton)

For the T1P aged samples, both in the warp and weft directions see Figure 12 (left), and the explanation in Supplementary Materials, Figure S5 (Detailed evolution of Aged samples T1P (Polycotton)).

For the T2P aged samples, both in the warp and weft directions see Figure 12 (right), and the explanation in Supplementary Materials, Figure S6 (Detailed evolution of Aged samples T2P (Polycotton)).

#### 3.3.7. Detailed Evolution of Aged Samples T3P Warp (Primed Linen)

The process is similar to that observed in the new T3P warp, previously explained in Section 3.3.2. If the tension in the aged T3 warp (canvas) is reduced from the beginning of its elastic phase (ε = 14.9% and 400 N) to 0 N, it contracts to 10.69%, following the slope of its elastic phase.

At the end of its elastic phase, the aged T3P warp reaches 1318 N at 17.64% elongation.

If the T3P warp reduces its elongation to 10.69%, the canvas reaches 0 N while the ground maintains 234.66 N. If the ground continues to release tension until it reaches 0 N, the T3P warp follows the slope of the ground’s elastic phase, and the elongation decreases to ε = 6.86%. At this point, the canvas—previously at 10.69% and 0 N—contracts and wrinkles. See Figure 13 (left).

#### 3.3.8. Detailed Evolution of Aged Samples T4P Warp (Primed Linen)

Just before the breaking point of the aged T4P warp (ε = 8.52%), if the tension in the T4 warp (canvas) is reduced to 0 N, it contracts to 8.31%. At this elongation, the T4 warp remains under slight tension. In the T4P warp, if the ground continues to contract from 690 N at 8.31% to ε = 1.35% and 0 N, the canvas contracts and wrinkles. See Figure 13 (right).

#### 3.3.9. Detailed Evolution of Aged Samples T3P andT4P Weft (Primed Linen)

In Figure 13 (left), the aged-T3P-weft (primed) and the aged-T3-weft (unprimed) elastic phases are very close and nearly intersect. In Figure 13 (right), the aged-T4P-weft (primed) and the aged-T4-weft (unprimed) elastic phases intersect This overlap prevents accurate quantification of the ground layer’s evolution during the dynamometric tests for these samples.

### 3.4. Ground Degradation

#### 3.4.1. Ground Degradation in Primed Linen

**Figure 14:**
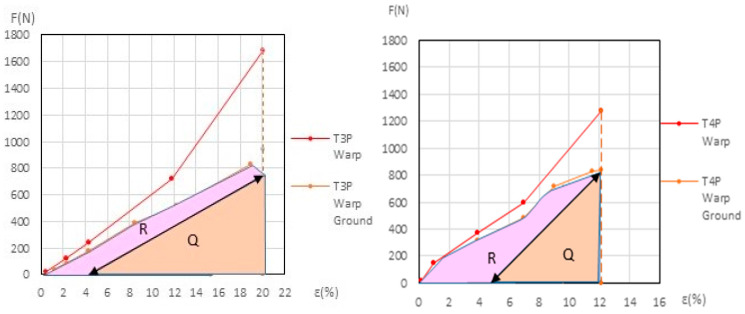
*R* and *Q* areas for new T3P warp (**left**) and new T4P warp (**right**) at maximum elastic elongation.

**Figure 15:**
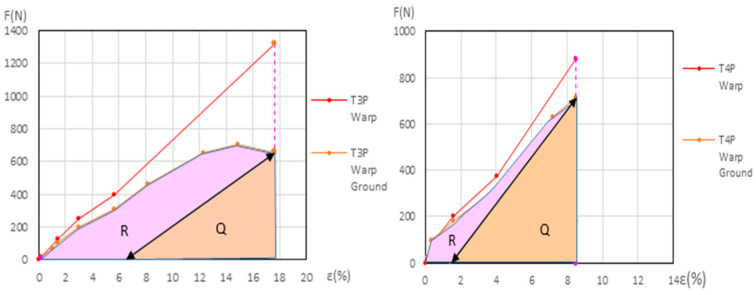
*R* and *Q* areas in the aged T3P warp (**left**) and aged T4P warp (**right**) at maximum elastic elongation.

For the primed linen samples T3P warp and T4P warp, both new and aged, the *Ground Degr%* and the ∆*% Aging* variables were calculated using the method described in Section 2.5, based on Equations (1)–(5) and shown in Tables S8–S11. The results are in Table 7 and in Table S12. The corresponding graphs are shown in Figure 14 (left and right) for the new samples and in Figure 15 (left and right) for the aged samples.

Table 7 shows the *Ground Degr%* and the ∆% *Aging* at the maximum elastic elongation in the linen warp samples, both new and aged. This table is also included as Table S12 in the Supplementary Materials. The *Ground Degradation%* in the linen weft direction could not be calculated due to the overlap or intersection of the force–elongation curves for the primed and unprimed samples.

#### 3.4.2. Ground Degradation in Primed Polycottons

**Figure 16:**
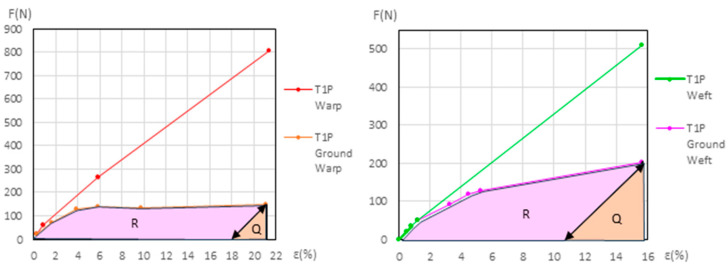
Maximum elongations for new T1P warp (**left**) and new T1P weft (**right**).

**Figure 17:**
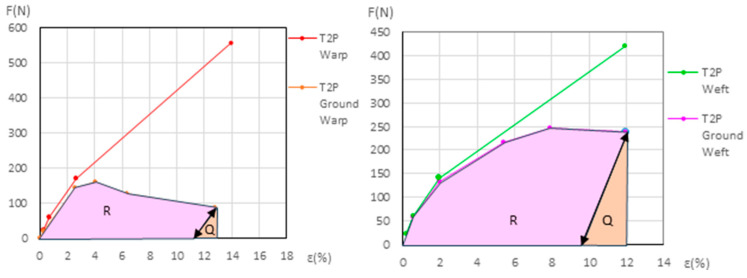
Maximum elongations for new T2P warp (**left**) and new T2P weft (**right**).

**Figure 18:**
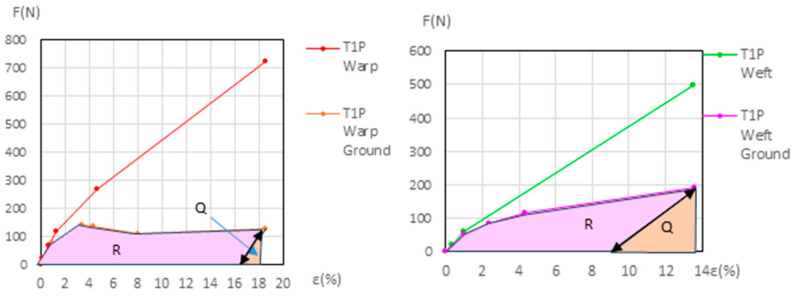
Maximum elongations for aged T1P warp (**left**) and aged T1P weft (**right**).

**Figure 19:**
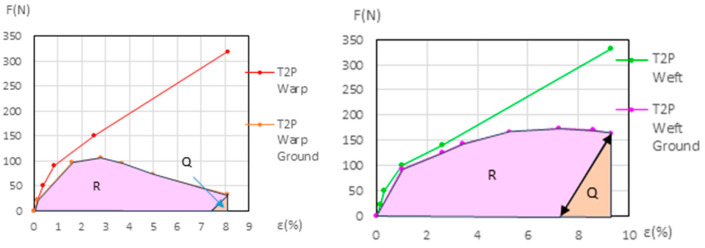
Maximum elongations for aged T2P warp (**left**) and aged T2P weft (**right**).

In the primed polycotton samples, the *Ground Degr (%)* and the ∆% *Aging* variables were also calculated using the method described in Section 2.5, with Equations (1)–(5), following the same procedure as used for the warp linen samples. These calculations, for both the new and aged conditions, are presented in Tables S13–S20. The results are in Table 8 and Table S21. The corresponding graphs are shown in Figure 16 and Figure 17 for the new samples, and in Figure 18 and Figure 19 for the aged samples. For these samples, the ground elongation at the end of the first cycle—when the force decreases to 0 *N*—is obtained descending from the ground elongation value at the end of the elastic third phase in the primed samples, following the ground-layer elastic slope during that phase.

Table 8 shows the *Ground Degr%* and the ∆*% Aging* values for the new and aged primed polycottons samples, calculated as explained before. This information is also included as Table S21 in the Supplementary Materials.

#### 3.4.3. Ground Degradation at the Bedding Down

Table S22 presents the *Ground bed Degr%* values for the new samples during the bedding-down sub-phase. This corresponds to the ratio between their N % area in the bedding down and their N *ε %* area in the first cycle, according to the calculation method described in Section 2.5. The resulting values are very small or zero. Table S23 shows the *Ground Degr%* for the aged samples during the bedding down, calculated using the same method.

### 3.5. Discussion

The obtained results reveal that in the studied samples, both primed and unprimed, the difference in elongation capacity between the canvas and the ground layers leads to deformation and degradation.

In polycottons, when stretched, the ground layer exhibits a short elastic phase with minimal elongation. Once it exceeds this elastic limit, it undergoes irreversible (plastic) deformation. The canvas, by contrast, has a much longer viscoelastic phase—exhibiting some permanent deformation, though not very significant—followed by an extended elastic phase.

If the ground layer is stretched beyond its elastic limit, it becomes permanently deformed, with deformation increasing as elongation increases, but it does not wrinkle at this stage. As the applied force decreases, the ground contracts and its inherent force returns to zero, yet the canvas still maintains force at this specific elongation. If the canvas force decreases to zero, its elongation also decreases, causing the ground to shrink further and develop irreversible plastic deformation and wrinkles.

In the case of primed linen samples (valid only for the warp direction), the opposite of polycottons occurs. Linen canvas exhibits a long viscoelastic phase characterized by low initial force and high elongation, followed by its elastic phase with a high slope. Immediately after the bedding-down phase, the ground initiates its elastic phase, characterized by a steep slope. From this point, as the force on the ground is reduced, its elongation drops below that of the canvas, causing the canvas to shrink and wrinkle.

The method for calculating the ground-layer degradation was developed and successfully applied to all samples providing valuable insights into their mechanical behavior. However, in the case of linen weft samples—where the weft direction generates the greatest force and causes their graph lines to overlap—this calculation was not possible.

In the new polycotton samples, the measured ground degradation reveals a notable difference between the warp and weft directions, with the weft exhibiting significantly less degradation than the warp. One factor that may explain the greater degradation in the warp direction is the high elongation observed in the T1P sample along that direction. However, it is important to recognize that the results obtained arise from a combination of interacting and non-linear factors. It should also be taken into account that the canvas surface is uneven due to the cylindrical threads and gaps in the weave, creating peaks and valleys. When a ground layer is applied, its thickness varies across these irregularities, affecting how it bonds with the fibers. This uneven bonding and surface texture imply that the overall properties of the canvas and ground are an average of many small variations rather than being uniform.

It should also be considered that in polycottons canvases, the warp and weft yarns often differ in both composition and structure. The warp yarns of T1 have a higher polyester content than T2, are more tightly spun, and are coated with sizing, giving them a smooth, defined cylindrical shape. In contrast, the weft yarns, both of T1 and T2, contain more cotton and are looser and hairier, with a less compact fiber structure. These differences affect the canvas surface texture, absorbency, and how the ground interacts with each direction of the weave—contributing to anisotropic behavior across the canvas.

In the case of linen, the degradation of the ground is much lower than in polycottons. However, aging still has a significant impact, especially in the case of T3, where degradation can reach up to 27%.

It should be noted that the ground layer on linen samples shows a much stronger hold than that on polycotton, as linen grounds are three to four times more resistant than polycotton grounds.

In the T3P and T4P warp samples, the ground holds much better throughout the first part of the canvas viscoelastic phase, holding better than the canvas itself. After, the trend reverses: the canvas continues to withstand stress, while the ground begins to degrade. One possible factor contributing to the lower degradation rate of linen compared to polycotton may be the more effective interaction and bonding between the ground layer and the linen fibers.

When the samples are aged, the behavior of T3P and T4P differs significantly.

It is worth noting the potential limitations of the study, as the results were obtained under exclusively uniaxial loading conditions and without the application of cyclic fatigue, which could be addressed in future research. It should also be considered that the parameters used for accelerated aging may have caused alterations in some of the acrylic resins present in the ground layers. As previously discussed, identifying effective yet safe aging conditions is particularly challenging when dealing with composite materials. While this study focuses on the mechanical aspects of degradation, we recognize the value of incorporating chemical transformation data—such as FTIR or colorimetric analyses—in future research to provide a more complete picture.

## 4. Conclusions

The canvases and ground layers of the eight selected samples—comprising two types of linen and two types of polycotton, each with and without a ground layer—were thoroughly identified and characterized.

Through dynamometric testing, the mechanical behavior of the samples was examined. The analysis of the behavior of canvas and ground materials revealed four distinct phases in their force–elongation response: the slack and bedding-down zone, viscoelastic response, elastic behavior, and material failure. Each phase reveals the distinct mechanical responses of the canvas and ground and their associated issues, such as crimp removal in the canvas and early brittle behavior in the ground, as well as shrinkage, wrinkling, and cracking culminating in irreversible deformation and fracture.

Understanding these responses helps explain how specific material properties contribute to conservation issues frequently observed in artworks, including shrinkage, wrinkling, and canvas and ground plastic deformation and cracking.

Using the same canvas samples with and without a ground layer allowed us to develop a method to calculate the mechanical ground degradation %, which helps to better understand how primed canvases and ground layers respond to tension. The method is based on calculating the ground graphic as the difference between both primed and unprimed graphics and can also be applied to similar cases, offering a useful tool for conservation professionals when assessing the condition and treatment needs of paintings.

## Figures and Tables

**Table 1:** Composition of yarn and canvas, canvas weight, and yarn spinning system for the four canvas samples.

Sample	Fiber Type Warp Threads	Fiber Type Weft Threads	Whole Canvas Composition	Canvas Weight g/m^2^	Threads/cm	Yarn Spinning System
T1	Polyester 66% Viscose 34%	Cotton 50.5% Polyester 35.5% Viscose 16%	Cotton 25%; Polyester 50%; Viscose 25%	168.20	Warp 18 Weft 11	open-end spinning
T2	Polyester 52.5% Viscose 47.5%	Cotton 71% Polyester 29% (Flax traces)	Cotton 60%; Polyester 40%;	157.99	Warp 13 Weft 13	open-end spinning
T3	Flax 100%	Flax 100%	Linen 100%	286.99	Warp 16.8 Weft 13.9	ring spinning
T4	Flax 100%	Flax 100%	Linen 100%	213.62	Warp 12.6 Weft 12.6	ring spinning

**Table 2:** Summary of the characteristics of the layers, fillers and pigments in each of the ground layers of the four samples.

Sample	Layer Number	Thickness (μ)	Pigments and Fillers	Optical Microscope Image. Cross Section (Objective MPlan10X/0.25)	Optical Microscope Image. Cross Section. UV Light (Objective MPlan10X/0.25)	SEM Image with Backscattered Electron Detector (BSE) Cross Section
T1P	4	30	Calcite, titanium white (rutile)	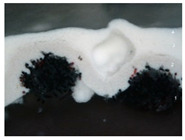	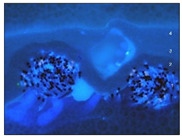	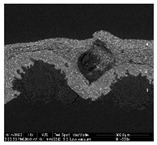
	3	45	Titanium white (rutile), calcite			
	2	35	Titanium white (rutile), calcite			
T2P	3	25	Dolomite, talc, titanium white (rutile) (l.p.), barium white (e.l.p.), calcium phosphate (e.l.p.)	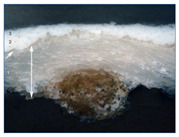	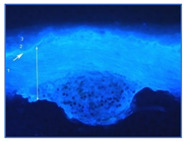	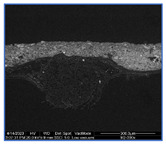
	2	30–65	Dolomite, talc, titanium white (rutile) (l.p.), barium white (e.l.p.)			
T3P	2	50–70	Calcite, titanium white (rutile) (l.p.)	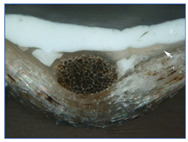	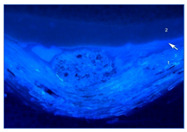	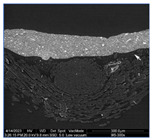
T4P	2	60–110	Dolomite, calcite (l.p.), titanium white (rutile) (l.p.), talc (v.l.p.)	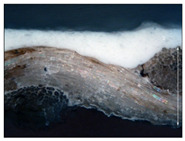	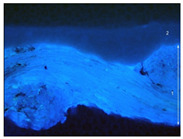	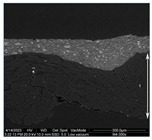

**Table 3:** Percentage composition of each pigment and filler found in the ground layers of each sample.

T1P	T2P	T3P	T4P
CaCO_3_ Calcite, syn (2.000) 55.8% TiO_2_ Rutile, syn (3.400) 44.2%	TiO_2_ Rutile syn (3.400) 14.7% CaMg(CO_3_)_2_ dolomite 53.7% Mg_3_Si_4_O_10_(OH)_2_ talc 31.6% BaSO_4_ baryte traces Calcium phosphate traces	CaCO_3_ calcite (3.150) 59.7% TiO_2_ rutile, syn (3.200) 40.3%	CaMg(CO_3_)_2_ dolomite ((1)) 80.4% TiO_2_ Rutile syn (3.400) 6.1% Mg_3_Si_4_O_10_(OH)_2_ talc 2M ((1)) 11.7% CaCO_3_ calcite, syn (2.000) 1.7%

**Table 4:** Dynamometric phases.

PHASES	1st PHASE	2nd PHASE	3rd PHASE	4th PHASE
**BEHAVIOUR**	ELASTIC Force/ elongation li-neal	VISCOELASTIC Force/ elongation not lineal	ELASTIC Force/ elongation lineal	PLASTIC Permanent deformation
***PHENOMENA***	*Slack + Bedding Down*	*Crimp removal*		*Erratic/sample break*
**MATERIAL** **AFFECTED**	**Canvas Unprimed**	**Canvas Unprimed**	**Canvas Unprimed** (at big ε)	**Canvas Unprimed**
**MATERIAL** **AFFECTED**	**Canvas Primed**	**Canvas Primed**	**Canvas Primed** (at big ε)	**Canvas Primed**
**MATERIAL** **AFFECTED**	**Ground**		**Ground** (at small ε)	**Ground**

**Table 5:** Force and elongation values for new samples at the different mechanical phases [4].

New (Not Aged) Samples Averaged Values *F*, *F/L*, *ε*, and *ε/ε_bed_* Ratio at the Different Phases													
Samples	1st Phase Up to the Bedding			1st Phase End		2nd Phase End		3rd Phase End		4th Phase Max F		Break Values	
	*F*	*F/L*	*ε_bed_*	*ε*	*ε/ε_bed_*	*F*	*ε*	*F*	*ε*	*F*	*ε*	*F*	*ε*
	*N*	*N/m*	*%*	*%*		*N*	*%*	*N*	*%*	*N*	*%*	*N*	*%*
T1 Warp	20	377	1.556	1.598	1.027	265	9.76	642	21.1	677	23.1	550	26.0
T2 Warp	20.0	377	2.520	2.540	1.008	170	6.36	431	12.8	447	13.7	262	14.7
T3 Warp	20.4	385	1.144	1.192	1.042	720	18.96	1314	22.0	1391	27.0	379	37.3
T4 Warp	20.1	379	1.672	1.716	1.026	375	11.57	1030	15.6	1124	16.3	290	22.2
T1 Weft	20.3	383	3.206	3.254	1.015	50	5.28	374	18.4	380	18.6	253	17.5
T2 Weft	20.4	385	5.400	5.424	1.004	140	10.56	303	16.3	363	19.9	77	23.9
T3 Weft	20.4	385	0.152	0.178	1.171	150	1.00	1276	4.08	1322	4.08	294	36.1
T4 Weft	22.0	415	0.308	0.382	1.240	300	2.20	1202	5.02	1277	5.50	284	17.0
T1P Warp	21.6	408	0.212	0.274	1.292	265	5.80	806	21.4	812	21.5	810	21.5
T2P Warp	24	453	0.230	0.28	1.217	170	2.64	557	13.9	557	13.9	538	14.1
T3P Warp	21	396	0.452	0.472	1.044	720	11.84	1686	20.1	1686	20.1	1608	20.2
T4P Warp	20.6	390	0.114	0.152	1.333	600	6.90	1280	12.2	1294	12.4	931	13.6
T1P Weft	21.4	404	0.460	0.522	1.135	50	1.24	509	15.7	509	15.7	501	15.7
T2P Weft	22	415	0.150	0.180	1.200	140	2.000	419	11.9	421	12.0	95	25.5
T3P Weft	21	396	0.152	0.166	1.092	150	0.66	1714	6.10	2007	7.43	1989	7.4
T4P Weft	29.0	547	0.170	0.203	1.191	300	1.70	1703	7.75	1703	7.75	1637	7.9

Note: *F* force (*N*); *ε* elongation or strain (*%*); *F/L* force/length (*N/m*); *ε _bed_* elongation increase at bedding down (*%*).

**Table 6:** Force and elongation values for aged samples at the different mechanical phases [4].

Aged Samples Averaged Values *F*, *F/L*, *ε*, and *ε/ε_bed_* Ratio at the Different Phases													
Samples	1st Phase Up to the Bedding			1st PHASE END		2nd Phase End		3rd Phase End		4th Phase Max F		Break Values	
	*F*	*F/L*	*ε_bed_*	*ε*	*ε/ε_bed_*	*F*	*ε*	*F*	*ε*	*F*	*ε*	*F*	*ε*
	*N*	*N/m*	*%*	*%*	*-*	*N*	*%*	*N*	*%*	*N*	*%*	*N*	*%*
T1 Warp	20.4	385	1.002	1.046	1.044	265	8.02	651	20.3	654	20.5	543	21.0
T2 Warp	20.8	392	1.452	1.614	1.112	150	4.98	347	9.42	402	11.4	209	12.8
T3 Warp	19.3	363	0.988	1.057	1.070	400	14.90	801	19.1	976	26.8	725	37.3
T4 Warp	21.0	396	1.828	1.912	1.046	375	10.50	882	13.5	943	14.1	212	23.1
T1 Weft	21.0	396	2.300	2.350	1.022	60	4.30	306	13.6	312	14.0	214	15.5
T2 Weft	20.1	379	3.370	3.420	1.015	140	8.58	322	13.4	363	15.2	142	17.6
T3 Weft	20.0	377	0.148	0.172	1.162	200	1.18	994	4.08	1270	9.4	279	19.0
T4 Weft	21.0	396	0.424	0.468	1.104	350	2.46	1050	4.50	1147	4.9	386	11.4
T1P Warp	22.5	425	0.160	0.200	1.250	265	4.70	720	18.5	720	18.5	720	18.5
T2P Warp	21.5	406	0.122	0.158	1.295	150	2.54	320	8.08	320	8.08	370	12.8
T3P Warp	20.3	383	0.158	0.174	1.101	400	5.68	1318	17.6	1482	20.3	1477	20.5
T4P Warp	22.4	423	0.072	0.106	1.472	375	4.04	879	8.52	879	8.52	840	9.7
T1P Weft	22.0	415	0.270	0.310	1.148	60	1.00	497	13.5	497	13.5	496	13.6
T2P Weft	21.1	398	0.100	0.144	1.440	140	2.60	331	9.3	331	9.3	172	2.2
T3P Weft	18.2	343	0.090	0.104	1.156	200	0.79	1212	4.76	1728	8.5	1717	8.6
T4P Weft	22.4	423	0.068	0.090	1.324	350	2.00	1304	5.73	1304	5.73	1333	5.9

Note: *F* force (*N*); *ε* elongation or strain (*%*); *F/L* force/length (*N/m*); *ε _bed_* elongation increase at the bedding down (%).

**Table 7:** *Ground Degr%* and ∆*% Aging* in linen warp, new and aged.

Samples	*Ground Degr %* in Linen Warp		
	New	Aged	∆*% Aging*
**T3P warp**	32	59.0	27
**T4P warp**	44.8	55.8	11

**Table 8:** *Ground Degr %* in primed polycottons T1P and T2P (new and aged).

Samples	Total *Ground Degr%* in Polycottons		
	New	Aged	∆*% Aging*
T1P Warp	90.9	95.6	4.7
T1P Weft	75.5	79.7	4.2
T2P Warp	95.2	99.3	4.1
T2P Weft	87.5	93	5.5

## Data Availability

The original contributions presented in this study are included in the article/Supplementary Materials. Further inquiries can be directed to the corresponding author.

## Supplementary Materials

Materials: The following supporting information can be downloaded at: https://www.mdpi.com/article/10.3390/ma18174041/s1, Table S1: Force and elongation values at the sub-phases of the 2nd phase for new samples; Table S2: Force and elongation values at the sub-phases of the 2nd phase for aged samples; Table S3: New unprimed samples force / elongation equations: F(N) = A j ·ε (%) + B j; Table S4: New primed samples force/elongation equations: F(N) = A j ·ε (%) + B j; Table S5: Aged unprimed samples force/elongation equations: F(N) = A j ·ε (%) + B j; Table S6: Aged primed samples force/elongation equations: F(N) = A j ·ε (%) + B j; Table S7: Example for the calculation method of coefficients A and B for New T1 Warp phases 2 and 3; Table S8: Ground degradation calculated for New T3P Warp; Table S9: Ground degradation and efficiency calculated for New T4P Warp; Table S10: Ground degradation and efficiency calculated for Aged T3P Warp; Table S11: Ground degradation calcu-lation for Aged T4P Warp; Table S12: Ground Degr% and ∆% Ageing in linen warp new and aged; Table S13: Ground degradation for New T1P Warp at maximum elongation; Table S14: Ground degradation for New T1P Weft at maximum elongation; Table S15: Ground degradation for New T2P Warp at maximum elongation; Table S16: Ground degradation for New T2P Weft at maximum elongation; Table S17: Ground degradation for Aged T1P Warp at maximum elongation; Table S18: Ground degradation for Aged T1P Weft at ·maximum elongation; Table S19: Ground degradation for Aged T2P Warp at maximum elongation; Table S20: Ground degradation for Aged T2P Weft at maximum elongation; Table S21: *Ground Degr %* in primed polycottons T1P and T2P (new and aged). Table S22: *Ground Degr %* at the Bedding Down in new primed samples; Table S23: *Ground Degr %* at Bedding down in aged primed samples; Figure S1: Bedding Down for new T2 warp (left) and for new T2P warp (right); Figure S2: Detailed New T1P Ground and T1 Canvas; Figure S3: Detailed New T2P Ground and T2 Canvas; Figure S4: Detailed New T4-T4P Warp. Ground and Canvas Warp with elastic decreasing to F = 0; Figure S5: Detailed Aged T1P Ground and T1 Canvas; Figure S6: Detailed Aged T2P Ground and T2 Canvas.
