# Supplementary material for: Canvas-Ground Interaction: A New Approach to Quantifying Ground Mechanical Degradation
Source: Materials (Basel). 2025 Aug 28;18(17):4041. doi: 10.3390/ma18174041 (PMC12429563; doi:10.3390/ma18174041)
Supplement: Supplementary file 1 [file materials-18-04041-s001.zip › materials-3720886-supplementary.pdf]

materials

MDPI

Supplementary materials.

Table S1. Force and elongation values at the sub-phases of the 2nd phase for new samples.

Samples	Forces and elongations at new samples subphases							
	1st phase end		2nd phase A end		2nd phase B end		2nd phase C end	
	F	*ε*	*F*	*ε*	*F*	*ε*	*F*	*ε*
	N	*%*	*N*	*%*	*N*	*%*	*N*	*%*
**T1 Warp**	20.0	1.598	60	3.92	265	9.76		
**T2 Warp**	20.0	2.540	60	4.07	170	6.36		
**T3 Warp**	20.4	1.192	120	8.5	240	12.06	720	18.96
**T4 Warp**	20.1	1.716	150	9.02	375	11.57		
**T1 Weft**	20.3	3.254	35	4.5	50	5.28		
**T2 Weft**	20.4	5.424	60	7.9	140	10.56		
**T3 Weft**	20.4	0.178	70	0.5	150	1		
**T4 Weft**	22.0	0.382	100	1.28	300	2.2		
**T1P Warp**	21.6	0.274	60	0.84	265	5.80		
**T2P Warp**	24	0.280	60	0.66	170	2.64		
**T3P Warp**	21.0	0.472	120	2.26	240	4.32	720	11.84
**T4P Warp**	20.7	0.152	150	0.96	375	3.88	600	6.94
**T1P Weft**	21.4	0.522	35	0.82	50	1.24		
**T2P Weft**	22.0	0.180	60	0.56	140	2.00		
**T3P Weft**	21.0	0.166	70	0.38	150	0.66	180	7.08
**T4P Weft**	29.0	0.203	100	0.37	300	1.70		

Table S2. Force and elongation values at the sub-phases of the 2nd phase for aged samples.

Samples	Forces and elongations at aged samples subphases							
	1st phase end		2nd phase A end		2nd phase B end		2nd phase C end	
	F	*ε*	*F*	*ε*	*F*	*ε*	*F*	*ε*
	N	%	*N*	%	*N*	%	*N*	%
**T1 Warp**	20.4	1.046	65	3.38	115	4.38	265	8.02
**T2 Warp**	20.8	1.614	50	2.78	90	3.70	150	4.98
**T3 Warp**	19.3	1.057	130	8.18	250	12.28	400	14.9
**T4 Warp**	21.0	1.912	100	7.18	200	8.86	375	10.5
**T1 Weft**	21.0	2.350	60	4.30				
**T2 Weft**	20.1	3.420	50	5.30	100	7.26	140	8.58
**T3 Weft**	20.0	0.172	50	0.37	100	0.72	200	1.18
**T4 Weft**	21.0	0.468	100	1.34	200	1.90	350	2.46
**T1P Warp**	22.5	0.200	65	0.64	115	1.35	265	4.70
**T2P Warp**	21.5	0.158	50	0.4	90	0.84	150	2.54
**T3P Warp**	20.3	0.174	130	1.38	250	3.00	400	5.68
**T4P Warp**	22.4	0.106	100	0.50	200	1.60	375	4.04
**T1P Weft**	22.0	0.310	60	1.00				
**T2P Weft**	21.1	0.144	50	0.32	100	1.02	140	2.60
**T3P Weft**	18.2	0.104	50	0.28	100	0.42	200	0.79
**T4P Weft**	22.4	0.090	100	0.32	200	1.00	350	2.00

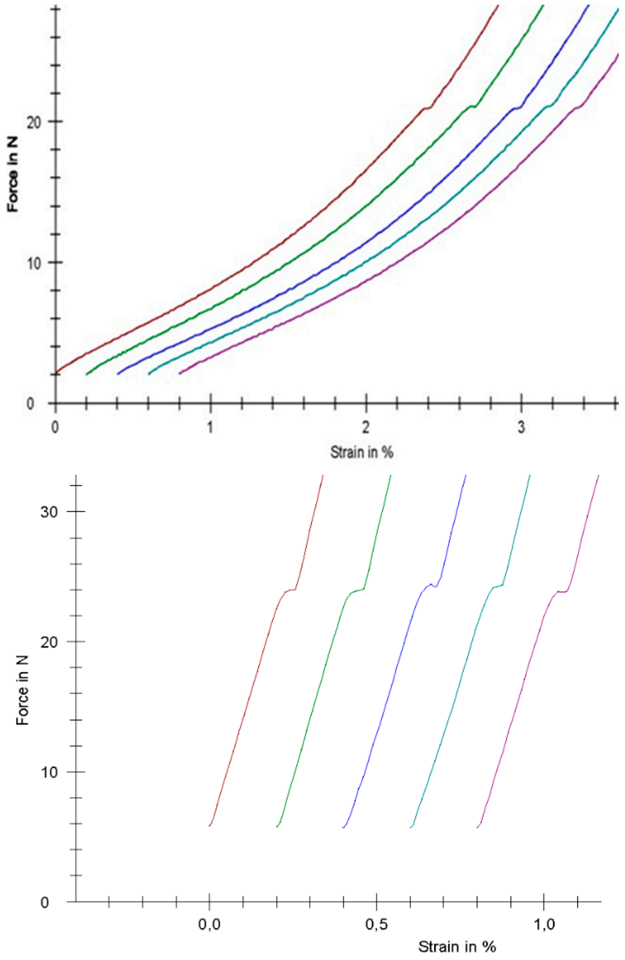

Figure S1. Bedding Down for new T2 warp (left) and for new T2P warp (right).

Table S3. New unprimed samples force / elongation equations: $F(N) = A_j \cdot \varepsilon\ (\%) + B_j$.

New unprimed samples force / elongation equations: $F(N) = A_j \cdot \varepsilon\ (\%) + B_j$											
**Samples and range**	**Phase Interval *j***	**ε range (%)**		**Coefficients**		**Samples and range**	**Phase Interval *j***	**ε range (%)**		**Coefficients**	
		**Down**	**Up**	A_j	B_j			**Down**	**Up**	A_j	B_j
**T1 Warp**	2-A	1.60	3.92	17.23	-7.528	**T1 Weft**	2-A	3.25	4.5	17.798	-18.090
**T1 Warp1**	2-B	3.92	9.76	35.1	-77.6	**T1 Weft1**	2-B	4.5	5.28	19.231	-51.538
**T1 Warp2**	3	9.76	21.10	33.25	-59.47	**T1 Weft2**	3	5.28	18.4	24.650	-80.154
**T2 Warp**	2-A	2.54	4.07	26.14	-46.41	**T2 Weft**	2-A	5.42	8.04	15.994	-66.349
**T2 Warp1**	2-B	4.07	6.36	48.04	-135.5	**T2 Weft1**	2-B	8.04	10.6	30.075	-177.6
**T2 Warp2**	3	6.36	12.4	40.528	-87.76	**T2 Weft2**	3	10.6	16.3	28.131	-157.1
**T4 Warp**	2-A	1.72	9.02	17.79	-10.44	**T4 Weft**	2-A	0.38	1.28	86.86	-11.18
**T4 Warp1**	2-B	9.02	11.6	88.24	-645.9	**T4 Weft1**	2-B	1.28	2.2	217.4	-178.3
**T4 Warp2**	2-C	11.57	13.1	147.1	-1326	**T4 Weft2**	3	2.2	5.02	319.9	-403.7
**T4 Warp3**	3	13.1	15.62	170.6	-1635	**T3 Weft**	2-A	0.18	0.5	154.04	-7.019
**T3 Warp**	2-A	1.192	8.5	13.629	4.15	**T3 Weft1**	2-B	0.5	1	160	-10
**T3 Warp1**	2-B	8.5	12.06	33.708	-166.5	**T3 Weft2**	3	1	4.08	366.02	-216.02
**T3 Warp2**	2-C	12.06	18.96	69.565	-599.0						
**T3 Warp3**	3	18.96	22	196.7	-3009						

Table-S4. New primed samples force / elongation equations: $F(N) = A_j \cdot \varepsilon\ (\%) + B_j$.

New primed samples force / elongation equations: $F(N) = A_j \cdot \varepsilon\ (\%) + B_j$											
**Samples and range**	**Phase - Interval *j***	**ε range (%)**		**Coefficients**		**Samples and range**	**Phase - Interval *j***	**ε range (%)**		**Coefficients**	
		**Down**	**Up**	A_j	B_j			**Down**	**Up**	A_j	B_j
**T1P Warp**	2-A	0.27	0.84	67.84	3.011	**T1P Weft**	2-A	0.52	0.82	45.638	-2.423
**T1P Warp1**	2-B	0.84	5.80	41.33	25.28	**T1P Weft1**	2-B	0.82	1.24	35.714	5.714
**T1P Warp2**	3	5.80	21.42	34.64	64.12	**T1P Weft2**	3	1.24	15.7	31.845	10.512
**T2P Warp**	2-A	0.28	0.66	94.74	-2.526	**T2P Weft**	2-A	0.18	0.56	100.00	4.000
**T2P Warp1**	2-B	0.66	2.64	55.56	23.33	**T2P Weft1**	2-B	0.56	2.00	55.556	28.889
**T2P Warp2**	3	2.64	13.95	34.227	79.642	**T2P Weft2**	3	2	11.95	28.009	83.983
**T4P Warp**	2-A	0.15	0.96	160.1	-3.671	**T4P Weft**	2-A	0.203	0.37	423.88	-56.84
**T4P Warp1**	2-B	0.96	3.88	77.05	76.03	**T4P Weft1**	2-B	0.37	1.7	150.38	44.361
**T4P Warp2**	2-C	3.88	6.94	73.53	89.71	**T4P Weft2**	3	1.7	7.75	231.94	-94.30
**T4P Warp3**	3	6.94	12.17	130	-302.2	**T3P Weft**	2-A	0.166	0.38	228.97	-17.009
**T3P Warp**	2-A	0.472	2.26	55.369	-5.134	**T3P Weft1**	2-B	0.38	0.66	285.71	-38.57
**T3P Warp1**	2-B	2.26	4.32	58.252	-11.65	**T3P Weft2**	3	0.66	6.1	287.50	-39.75
**T3P Warp2**	2-C	4.32	11.84	63.830	-35.74						
**T3P Warp3**	3	11.84	20.1	116.9	-664.6						

Table S5. Aged unprimed samples force / elongation equations: $F(N) = A_j \cdot \varepsilon\ (\%) + B_j$.

Aged unprimed samples force / elongation equations: $F(N) = A_j \cdot \varepsilon\ (\%) + B_j$											
**Samples and range**	**Phase - Interval *j***	**ε range (%)**		**Coefficients**		**Samples and range**	**Phase Interval j**	**ε range (%)**		**Coefficients**	
		**Down**	**Up**	A_j	B_j			**Down**	**Up**	A_j	B_j
**T1 Warp**	2-A	1.046	3.38	-0.043	65.145	**T1 Weft**	2-A	2.35	4.3	19.49	-23.790
**T1 Warp1**	2-B	3.38	4.38	50.00	-104.00	**T1 Weft1**	3	4.3	13.6	26.57	-54.233
**T1 Warp2**	2-C	4.38	8.02	41.209	-65.495	**T2 Weft**	2-A	3.42	5.3	15.92	-34.349
**T1 Warp3**	3	8.02	20.29	31.479	12.541	**T2 Weft1**	2-B	5.3	7.26	25.51	-85.204
**T2 Warp**	2-A	1.614	2.78	25.043	-19.619	**T2 Weft2**	2-C	7.26	8.58	30.30	-120.00
**T2 Warp1**	2-B	2.78	3.7	43.478	-70.870	**T2 Weft3**	3	8.58	13.4	37.45	-181.31
**T2 Warp2**	2-C	3.7	4.98	46.875	-83.437	**T4 Weft**	2-A	0.47	1.34	90.60	-21.40
**T2 Warp3**	3	4.98	9.42	44.414	-71.184	**T4 Weft1**	2-B	1.34	1.9	178.5	-139.29
**T4 Warp**	2-A	1.912	7.18	14.996	-7.6727	**T4 Weft2**	2-C	1.9	2.46	267.8	-308.93
**T4 Warp1**	2-B	7.18	8.86	59.524	-327.381	**T4 Weft3**	3	2.46	4.5	343.1	-494.12
**T4 Warp2**	2-C	8.86	10.5	106.71	-745.427	**T3 Weft**	2-A	0.172	0.37	151.5	-6.06
**T4 Warp3**	3	10.5	13.46	171.28	-1423.5	**T3 Weft1**	2-B	0.37	0.72	142.8	-2.86
**T3 Warp**	2-A	1.057	8.18	15.548	2.8178	**T3 Weft2**	2-C	0.72	1.18	217.3	-56.52
**T3 Warp1**	2-B	8.18	12.28	29.268	-109.415	**T3 Weft3**	3	1.18	4.08	273.7	-123.08
**T3 Warp2**	2-C	12.28	14.90	57.252	-453.053						
**T3 Warp3**	3	14.90	19.13	94.97	-1015.1						

Table S6. Aged primed samples force / elongation equations: $F(N) = A_j \cdot \varepsilon\ (\%) + B_j$.

Aged primed samples force / elongation equations: $F(N) = A_j \cdot \varepsilon\ (\%) + B_j$											
**Samples and range**	**Phase - Interval j**	**ε range (%)**		**Coefficients**		**Samples and range**	**Phase - Interval j**	**ε range (%)**		**Coefficients**	
		**Down**	**Up**	A_j	B_j			**Down**	**Up**	A_j	B_j
**T1P Warp**	2-A	0.200	0.64	96.591	3.182	**T1P Weft**	2-A	2.35	1	55.07	4.928
**T1P Warp1**	2-B	0.64	1.35	70.423	19.93	**T1P Weft1**	3	1	13.5	34.83	25.170
**T1P Warp2**	2-C	1.35	4.70	44.776	54.552	**T2P Weft**	2-A	0.14	0.32	164.20	-2.545

**T1P Warp3**	3	4.70	18.53	32.896	110.388	**T2P Weft1**	2-B	0.32	1.02	71.429	27.143
**T2P Warp**	2-A	0.158	0.4	117.769	2.983	**T2P Weft2**	2-C	1.02	2.6	25.316	74.177
**T2P Warp1**	2-B	0.4	0.84	90.909	13.636	**T2P Weft3**	3	2.6	9.3	28.460	66.003
**T2P Warp2**	2-C	0.84	2.54	35.294	60.353	**T4P Weft**	2-A	0.09	0.32	337.39	-7.965
**T2P Warp3**	3	2.54	8.08	30.386	72.058	**T4P Weft1**	2-B	0.32	1.00	147.06	52.941
**T4P Warp**	2-A	0.106	0.5	196.954	1.523	**T4P Weft2**	2-C	1.00	2.00	150.00	50.000
**T4 Warp1**	2-B	0.5	1.6	90.909	54.545	**T4P Weft3**	3	2.00	5.73	256.09	-162.173
**T4P Warp2**	2-C	1.6	4.04	71.72	85.25	**T3P Weft**	2-A	0.104	0.28	180.68	-0.5909
**T4P Warp3**	3	4.04	8.52	112.50	-79.50	**T3P Weft1**	2-B	0.28	0.42	357.14	-50.00
**T3P Warp**	2-A	0.174	1.38	90.962	4.4726	**T3P Weft2**	2-C	0.42	0.79	270.27	-13.514
**T3P Warp1**	2-B	1.38	3	74.074	27.778	**T3P Weft3**	3	0.79	4.76	254.91	-1.380
**T3P Warp2**	2-C	3.00	5.68	55.97	82.090						
**T3P Warp3**	3	5.68	17.64	76.756	-35.97						

Table S7. Example for the calculation method of coefficients A and B for New T1 Warp phases 2 and 3.

**Coefficients and calculations for New T1 Warp, T1 Warp1 and T1 Warp2 equations**							
**ε(%)**	**F(N)**	Phase	**Coefficients**		**A and B coefficients calculations according the F(N) and ε(%) values at 2nd and 3rd phases**	**Linear equations** ***F = A $_j$ * ε + B $_j$***	
**1,598**	**20,0**		***A $_j$***	***B $_j$***			
**3,92**	**60**	2-A	**17,227**	**-7,528**	*A $_j$* = (60-20) / (3,92-1,598) ; *B $_j$* = (3,92*20-60*1,598) / (3,92-1,598)	**y = 17,227 x - 7,528**	**T1 Warp**
**9,76**	**265**	2-B	**35,103**	**-77,603**	*A $_j$* = (265-60) / (9,76-3,92) ; *B $_j$* = (9,76*60-265*3,92) / (9,76-3,92)	**y = 35.10 x - 77.60**	**T1 Warp1**
**21,1**	**642**	3	**33,245**	**-59,473**	*A $_j$* = (642-265) / (21,1-9,76); *B $_j$* = (21,1*265-642*9,76) / (21,1-9,76)	**y = 33.245 x - 59.473**	**T1 Warp2**

Table S8. Ground degradation calculated for New T3P Warp.

**New T3P Warp Ground 1st cycle**			**New T3P Warp Ground 2nd cycle**				
**Elong**	**Force**	**Area *F* · *ε***	**Elong**	***Δ* Elong**	**Force**	**Area *F* · *Δε***	
***ε*(%)**	***F*(N)**	***N* · %**	***ε*(%)**	***Δε*(%)**	***F*(N)**	***N* · %**	
0.178	20.4		4.17		0		
1.192	40.8	31.0	20.1		741.8		
2.26	85.0	67.2		15.93	741.8	**5908**	**Total Q**
4.32	177.0	269.9			*Ground Eff %*	**68.0**	
8.5	386.8	1178.3			*Ground Degr %*	**32.0**	**2nd cycle**
12.28	521.4	1716.5					
18.96	832.6	4522.4					
20.1	741.8	897.4					
	**Total Q+R**	**8683**					

The 1st cycle corresponds to the ground area of the first elongation up to the end of sample elongation. The 2nd cycle is the ground triangle area corresponding at the *Δ* elongation after the force decrease to 0 newtons and the force increase up to the maximum force attained before. In the linen new samples, the *Ground Eff%* and the *Ground Degr%* are:

$$\text{Ground Eff \%} = 100 \cdot \text{Total Q} / \text{Total (Q+R)} \text{ and Ground Degr \%} = 100 - \text{Ground Eff \%} \quad (1)$$

Table S9. Ground degradation and efficiency calculated for New T4P Warp.

New T4P Warp Ground 1st cycle			New T4P Warp Ground 2nd cycle				
**Elong**	**Force**	**Area F · ε**	**Elong**	***Δ* Elong**	**Force**	**Area F · ε**	
***ε*(%)**	***F*(N)**	**N · %**	***ε*(%)**	***Δε*(%)**	***F*(N)**	**N · %**	
0.15	20		4.7		0		
1.716	188.2	163.0	12.17		838.17		
3.88	316.4	546.0		7.47	838.17	**3130.6**	**Total Q**
6,.94	487.0	1229.2			*Ground Eff %*	**55.2**	
9.02	720.6	1255.9			*Ground Degr %*	**44.8**	**2nd cycle**
11.57	827.1	1973.3					
12.17	838.7	499.7					
	**Total Q+R**	**5667.1**					

The cycles are the same, as in the Table S8.

Table S10. Ground degradation and efficiency calculated for Aged T3P Warp.

T3P Warp Ground Aged 1st cycle			T3P Warp Ground Aged 2nd cycle				
**Elong**	**Force**	**Area *F · ε***	**Elong**	***Δ* Elong**	**Force**	**Area *F · ε***	
***ε*(%)**	***F*(N)**	***N · %***	***ε*(%)**	***Δε*(%)**	***F*(N)**	***N · %***	
0	5		6.86		0		
0.174	20.3		17.64		660		
1.057	72.4	40.9		10.78	660	**3557.4**	**Total Q**
1.38	105.7	28.8			*Ground Eff %*	**45.2**	
3	200.5	248.1			*Ground Degr %*	**54.8**	**2nd cycle**
5.68	308.9	682.6			*100 (1-(3557 / 7887)) =*	*54.8*	
8.18	461.9	963.5			**Total Q+R new (N*%)**	**8683**	**1st cycle**
12.28	656.6	2292.9			***Total Ground Degr %***	**59.0**	**2nd cycle**
14.9	707.7	1787.2			*100 (1-(3557 / 8683)) =*	*59.0*	
17.64	660.0	1873.7					
**Total Q+R Aged**		**7877**					

The cycles are the same as in the Table S8.

The *Total Ground Degr % = 100 · [1 - / (Total Q / Total (Q+R) new (N*%))]* at 2nd cycle (2)

Table S11. Ground degradation calculation for Aged T4P Warp.

T4P Warp Ground Aged 1st cycle			T4P Warp Ground Aged 2nd cycle				
**Elong**	**Force**	**Area F · ε**	**Elong**	***Δ* Elong**	**Force**	**Area F · ε**	
***ε*(%)**	***F*(N)**	**N · %**	***ε*(%)**	***Δε*(%)**	***F*(N)**	**N · %**	
0	5		1.35		0		
0.106	22.4		8.52		699.0		
0.320	96.5	12.7		7.17	699.0	**2506**	
1.6	183.7	179.3			*Ground efficiency %*	**85.9**	
4.04	322.1	617.1			*Ground degradation %*	**14.1**	**2nd cycle**
5.68	308.9	517.4			*100 (1-(2506 / 2919)) =*	*14.1*	
7.18	628.3	702.9			**Total Q+R new (N*%)**	**5667**	**1st cycle**
8.52	699.0	889.3			***Total Ground degr %***	**55.8**	**2nd cycle**

**Total Q+R Aged**	**2919**	*100 (1-(2506 / 5667)) =*	*55.8*

The cycles are the same as in the Table S8.

The *Total Ground Degr %* is calculated as in the Table S10.

Table S12. *Ground Degr%* and *Δ% Ageing* in linen warp new and aged.

Samples	***Ground Degr %*** **in linen warp**		
	**New**	**Aged**	*Δ% Ageing*
**T3P warp**	32	59,0	27
**T4P warp**	44.8	55,8	11

To facilitate and to understand the calculations, the Table 7 of the article is also included in the Table S12-of this Supplementary Material.

Table S13. Ground degradation for New T1P Warp at maximum elongation.

**New T1P Warp Ground 1st cycle**			**New T1P Warp Ground 2nd cycle**				
**Elong**	**Force**	**Area F · ε/2**	**Elong**	**Δ elong**	**Force**	**Area F · ε /2**	
**ε(%)**	**F(N)**	**N · %**	**ε(%)**		**F(N)**	**N · %**	
0,27	21.6		17.8		0		
1,60	71.3	61.7	21.1		147.2		
3,92	12.,3	230.6		3.3	147.2	**242.9**	**Total Q**
5,8	139	250.3			*Ground Eff %*	**9.1**	
9,76	134.5	541.5			*Total Ground Degr %*	**90.9**	**2nd cycle**
21,1	147.2	1597.2					
**Total P= Q+R**		**2681.4**					

The 1st cycle corresponds to the ground area of the first elongation up to the end of sample elongation. The 2nd cycle is the ground triangle area corresponding at the *Δ* elongation after the force decrease to 0 newtons and the force increase up to the maximum force attained before. In the Polycotton new samples the *Ground Eff%* and the *Ground Degr%* are:

Ground Eff % = 100 · Total Q / Total (Q+R) and *Ground Degr % = 100 – Ground Eff %* (3)

Table S14. Ground degradation for New T1P Weft at maximum elongation.

**New T1P Weft Ground 1st cycle**			**New T1P Weft Ground 2nd cycle**				
**Elong**	**Force**	**Area F · ε**	**Elong**	**Δ elong**	**Force**	**Area F · ε**	
**ε(%)**	**F(N)**	**N · %**	**ε(%)**		**F(N)**	**N · %**	
0,52	21.4		10.55		0		
1,24	50.0	25.7	15.66		203.4		
3,25	93.3	144.0		5.11	203.4	**519.7**	**Total Q**
4,5	118.8	132.6			*Ground Eff %*	**24.5**	
5,28	128.7	96.5			*Total Ground Degr %*	**75.5**	**2nd cycle**
15,66	203.4	1723.6					
	**Total Q+R**	**2122.4**					

The *Ground Eff %* and the *Ground Degr %* are calculated as in the Table S13.

Table S15. Ground degradation for New T2P Warp at maximum elongation.

**New T2P Warp Ground 1st cycle**			**New T2P Warp Ground 2nd cycle**			
**Elong**	**Force**	**Area F · ε**	**Elong**	**Δ elong**	**Force**	**Area F · ε**

ε(%)	F(N)	N · %	ε(%)		F(N)	N · %	
0,28	21.3		11.21		0		
2,54	144.4	187.2	12.8		86.7		
4,08	158.9	233.5		1.59	86.70	**68.9**	**Total Q**
6,36	127.3	326.3			***Ground Eff %***	**4.8**	
12,8	86.7	689.1			***Total Ground Degr %***	**95.2**	**2nd cycle**
	**Total Q+R**	**1436.1**					

The *Ground Eff* % and the *Ground Degr* % are calculated as in the Table S13.

Table S16. Ground degradation for New T2P Weft at maximum elongation.

**New T2P Weft Ground 1st cycle**			**New T2P Weft Ground 2nd cycle**				
**Elong**	**Force**	**Area F · ε**	**Elong**	**Δ elong**	**Force**	**Area F · ε**	
**ε(%)**	**F(N)**	**N · %**	**ε(%)**		**F(N)**	**N · %**	
0.18	22		9.55		0		
0.56	60.0	15.6	11.95		239.5		
2.00	132.7	138.7		2.4	239.50	**287.4**	**Total Q**
5.42	215.5	595.4			***Ground Eff %***	**12.5**	
7.9	245.3	571.4			***Total Ground Degr %***	**87.5**	**2nd cycle**
11.95	239.5	981.7					
	**Total Q+R**	**2302.9**					

The *Ground Eff* % and the *Ground Degr* % are calculated as in the Table ~~S8~~ S13.

Table S17. Ground degradation for Aged T1P Warp at maximum elongation.

**Aged T1P Warp Ground 1st cycle**			**Aged T1P Warp Ground 2nd cycle**				
**Elong**	**Force**	**Area F*ε**	**Elong**	**Δ elong**	**Force**	**Area F*Δε**	
**ε(%)**	**F(N)**	**N*%**	**ε(%)**		**F(N)**	**N*%**	
0,2	22.5		16.62		0		
1,046	73.2	40.5	18.53		124.13		
3,38	140.9	249.8		1.91	124.13	**118.5**	**Total Q**
4,38	135.7	138.3			***Ground Eff %***	**5.6**	
8,02	109.2	445.7			***Ground Degr %***	**94.4**	**2nd cycle**
18,53	124.1	1226.3			*100 (1-(124 / 2100)) =*	*94.4*	
**Total Q+R aged**		**2100,5**	**1st cycle**		**Total Q+R new (N*%)**	**2681.4**	**1st cycle**
					Total Ground Degr %	**95.6**	**2nd cycle**
					100 (1-(124 / 2681)) =	*95.6*	

The Total Ground Degr % = 100 · [1 - / (Total Q / Total (Q+R) new (N*%))] at 2nd cycle.

The cycles are the same as in the Table S13, and the *Total Ground Degr %* is calculated as in the Table S8.

Table 18. Ground degradation for Aged T1P Weft at ·maximum elongation.

**Aged T1P Weft Ground 1st cycle**			**Aged T1P Weft Ground 2nd cycle**				
**Elong**	**Force**	**Area F*ε**	**Elong**	**Δ elong**	**Force**	**Area F*Δε**	
**ε(%)**	**F(N)**	**N*%**	**ε(%)**		**F(N)**	**N*%**	
0.31	22		9.07		0		
1	51.1	25.2	13.6		190.6		
2.35	86.0	92.5		4.53	190.6	**431.7**	**Total Q**
4.3	114.9	195.9			***Ground Eff %***	**24.9**	

13.6	190.6	1420.8			***Ground Degr*** **%**	**75.1**	**2nd cycle**
**Total Q+R aged**		**1734.4**	**1st cycle**		*100 (1-(432 / 1734)) =*	*75.1*	
					**Total Q+R new (N*%)**	**2122.4**	**1st cycle**
					Total Ground Degr **%**	**79.7**	**2nd cycle**
					100 (1-(432 / 2122)) =	*79.7*	

The cycles are the same as in the Table S13, and the *Total Ground Degr %* is calculated as in the Table S8.

Table S19. Ground degradation for Aged T2P Warp at maximum elongation.

**Aged T2P Warp Ground 1st cycle**			**Aged T2P Warp Ground 2nd cycle**				
**Elong**	**Force**	**Area F*ε**	**Elong**	**Δ elong**	**Force**	**Area F*Δε**	
**ε(%)**	**F(N)**	**N*%**	**ε(%)**		**F(N)**	**N*%**	
0.16	21.5		7.46		0		
1.61	97.2	86.2	8.08		32.3		
2.78	106.5	119.2		0.62	32,3	**10.0**	**Total Q**
3.7	90	90.4			***Ground Eff %***	**98.2**	
4.98	73.4	104.6			***Ground Degr %***	**1.1**	**2nd cycle**
8.08	32.3	163.8			*100 (1-(10 / 564)) =*	*98.2*	
**Total Q+R aged**		**564.1**	**1st cycle**		**Total Q+R new (N*%)**	**1436.1**	**1st cycle**
					Total Ground Degr **%**	**99.3**	**2nd cycle**
					100 (1-(10 / 1436)) =	*99.3*	

The cycles are the same as in the Table S13, and the *Total Ground Degr %* is calculated as in the Table S8.

Table S20. Ground degradation for Aged T2P Weft at maximum elongation.

**Aged T2P Weft Ground 1st cycle**			**Aged T2P Weft Ground 2nd cycle**				
**Elong**	**Force**	**Area F*ε**	**Elong**	**Δ elong**	**Force**	**Area F*Δε**	
**ε(%)**	**F(N)**	**N*%**	**ε(%)**		**F(N)**	**N*%**	
**0.144**	**21.1**		**7.33**		**0**		
**1.02**	**94.0**	**50.4**	**9.3**		**163.7**		
**2.6**	**124.7**	**172.8**		**1.97**	**163.70**	**161.2**	**Total Q**
**3.42**	**143.2**	**109.8**			***Ground Eff %***	**12.4**	
**5.3**	**166.8**	**291.4**			***Ground Degr %***	**87.6**	**2nd cycle**
**7.26**	**172.6**	**332.6**			*100 (1-(161 / 1303)) =*	**87.6**	
**8.58**	**170.2**	**226.2**			**Total Q+R new (N*%)**	**2302.9**	**1st cycle**
**9.3**	**163.7**	**120.2**			***Total Ground Degr %***	**93.0**	**2nd cycle**
**Total Q+R aged**		**1303.5**	**1st cycle**		*100 (1-(161 / 2303)) =*	*93.0*	

The cycles are the same as in the Table S13, and the *Total Ground Degr %* is calculated as in the Table S8.

Table S21. *Ground Degr %* in primed polycottons T1P and T2P (new and aged).

Samples	**Total *Ground degr%* in polycottons**		
	**New**	**Aged**	***Δ% Ageing***
T1P Warp	90.9	95.6	4.7
T1P Weft	75.5	79.7	4.2
T2P Warp	95.2	99.3	4.1
T2P Weft	87.5	93	5.5

To facilitate and to understand the calculations, the article Table 8 is also included in this Supplementary Material as.Table S21.

Table S22. *Ground Degr %* at the Bedding Down in new primed samples.

New Samples	1st phase						
	Force	Bedding start	Bedding end	Canvas		Ground	Ground Degrad
				Unprimed	Primed		
	N	%	%	N · %	N · %	N · %	%
T1 Warp	20.0	1.556	1.598	0.840			
T2 Warp	20.0	2.52	2.540	0.400			
T3 Warp	20.4	1.144	1.192	0.979			
T4 Warp	20.08	1.672	1.716	0.884			
T1 Weft	20.3	3.206	3.254	0.974			
T2 Weft	20.4	5.400	5.424	0.490			
T3 Weft	20.4	0.152	0.178	0.530			
T4 Weft	22.0	0.308	0.382	1.628			
T1P Warp	21.6	0.212	0.274		1.339	0.499	**0.019**
T2P Warp	24	0.23	0.28		1.200	0.800	**0.056**
T3P Warp	21	0.452	0.472		0.420	-0.559	**0.000**
T4P Warp	20.66	0.114	0.152		0.785	-0.098	**0.000**
T1P Weft	21.4	0.460	0.522		1.327	0.352	**0.038**
T2P Weft	22	0.150	0.180		0.660	0.170	**0.007**
T3P Weft	21	0.152	0.166		0.294	-0.236	**0.000**
T4P Weft	29.0	0.170	0.203		0.943	-0.686	**0.000**

Table S22 shows the new samples *Ground Degr%* at the Bedding Down, comparing their N · % area in the Bedding Down with their N · % area in the 1st cycle, according the calculation method explained in Material and Methods. The values are very small or zero.

Table S23. *Ground Degr %* at Bedding down in aged primed samples.

Aged Samples	1st phase						
	Force	Bedding start	Bedding end	Canvas		Ground	Ground Degrad
				Unprimed	Primed		
	N	%	%	N · %	N · %	N · %	%
T1 Warp	20.4	1.002	1.046	0.898			
T2 Warp	22.5	0.160	0.200	0.900			
T3 Warp	21.0	2.300	2.350	1.050			
T4 Warp	22.0	0.270	0.310	0.880			
T1 Weft	20.8	1.452	1.614	3.370			
T2 Weft	21.5	0.122	0.158	0.774			
T3 Weft	20.1	3.370	3.420	1.004			
T4 Weft	21.1	0.100	0.144	0.928			
T1P Warp	19.3	0.9875	1.056		1.335	0.438	0.021
T2P Warp	20.3	0.158	0.174		0.325	-0.575	0.000
T3P Warp	20.0	0.148	0.172		0.480	-0.570	0.000
T4P Warp	18.2	0.090	0.104		0.255	-0.625	0.000
T1P Weft	21.0	1.828	1.912		1.764	-1.606	0.000
T2P Weft	22.4	0.072	0.106		0.762	-0.012	0.000
T3P Weft	21.0	0.424	0.468		0.924	-0.080	0.000
T4P Weft	22.4	0.068	0.090		0.493	-0.436	0.000

Table S23 shows the aged samples *Ground Degr%* at the Bedding Down, comparing their N · % area in the Bedding Down with their N · % area in the 1st cycle, according the calculation method explained at Material and Methods. The values are very small or zero.

Samples detailed evolution.

As Supplementary Materials we include here the detailed evolution of the new and aged Polycotton samples, except the new warp Polycotton samples which is in the main text. Here is included, also, the detailed evolution of the new T4P Warp linen samples.

Detailed evolution of New samples T1P Weft (Polycotton)

At the end of the elastic 3rd phase, the T1 weft (canvas) reaches a force of 374.4 N at an elongation of 18.44%. At this same phase endpoint, the T1P weft reaches 509.3 N at 15.66% elongation. At 15.66% elongation, the ground layer carries a force of 203.4 N. If the T1P weft begins to contract, the ground also contracts, following the slope of its elastic phase, from 50 N to 20 N, and attains the force 0 N at an elongation of ε = 10.55%. At this point (ε = 10.55%), the canvas is still under tension, while the ground is not. If the T1P weft continues to shrink, the canvas follows the same F/ε slope as the T1 weft in its elastic phase, and the force reaches 0 N at an elongation of ε = 3.25%. Between ε = 10.55% and ε = 3.25%, the ground continues to contract and wrinkle, resulting in plastic deformation. See here Figure S2, and in the main article the Fig 6 (left) and Fig 10 (left).

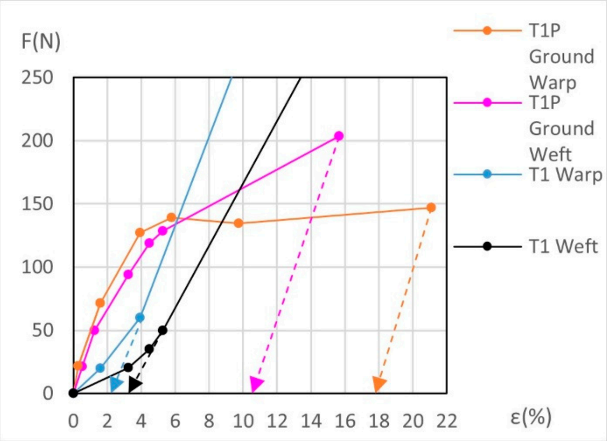

Figure S2. Detailed New T1P Ground and T1 Canvas.

Detailed evolution of New samples T2P Warp and Weft (Polycotton)

At the end of the elastic 3rd phase, the T2 warp (canvas) reaches a force of 431 N at an elongation of 12.8%. At this same phase endpoint, the T2P warp reaches 557 N at 13.95% elongation. At 12.8% elongation, the ground layer carries a force of 86.7 N. If the T2P warp begins to contract, the ground also contracts, following the slope of its elastic phase, from 144.4 N to 20 N, and attains the force 0 N at an elongation of ε = 11.21%. At this point (ε = 11.21%), the canvas is still under tension, while the ground is not. If the T2P warp continues to shrink, the canvas follows the same F/ε slope as the T2 warp in its elastic phase, and the force reaches 0 N at an elongation of ε = 2.82%. Between ε = 11.21% and ε = 2.82%, the ground continues to contract and wrinkle, resulting in plastic deformation. See here Figure S3, Fig. 6 (right) and Fig. 10 (right).

At the end of the elastic 3rd phase, the T2 weft (canvas) reaches a force of 302.6 N at an elongation of 16.34%. At this same phase endpoint, the T2P weft reaches 418.6 N at 11.95% elongation. At 11.95% elongation, the ground layer carries a force of 239.5 N. If the T2P weft begins to contract, the ground also contracts, following the slope of its elastic

phase, from 57.9 N to 20 N, and attains the force 0 N at an elongation of ε = 9.55%. At this point (ε = 9.55%), the canvas is still under tension, while the ground is not. If the T2P weft continues to shrink, the canvas follows the same F/ε slope as the T2 weft in its elastic phase, and the force reaches 0 N at an elongation of ε = 5.91%. Between ε = 9.55% and ε = 5.91%, the ground continues to contract and wrinkle, resulting in plastic deformation. See here Figure S3, and in the main article the Fig 6 (right) and Fig 10 (right).

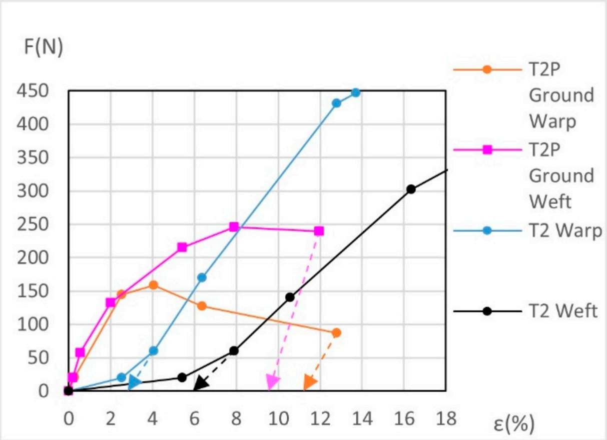

Figure S3. Detailed New T2P Ground and T2 Canvas.

Detailed evolution of New Samples T4P Warp (Linen)

If the tension in the T4 warp (canvas) is reduced from the beginning of its elastic phase (ε = 13.1% and 600 N) to 0 N, it contracts to 9.6%, following the slope of its elastic phase. At the end of its elastic phase, the T4P warp reaches 1280 N at 12.17% elongation. If the T4P warp and the ground layer then reduce their elongation to 9.6%, both reduce their tension to 550.1 N, as the canvas is at 0 N. If the T4P warp and ground continue to release tension until reaching 0 N, they follow the slope of the ground's elastic phase, and the elongation decreases to ε = 4.7%. At this point, the canvas—which was at 9.6% and 0 N—contracts and wrinkles, resulting in plastic deformation. See here Figure S4 and in the main article the Figure 11 (right).

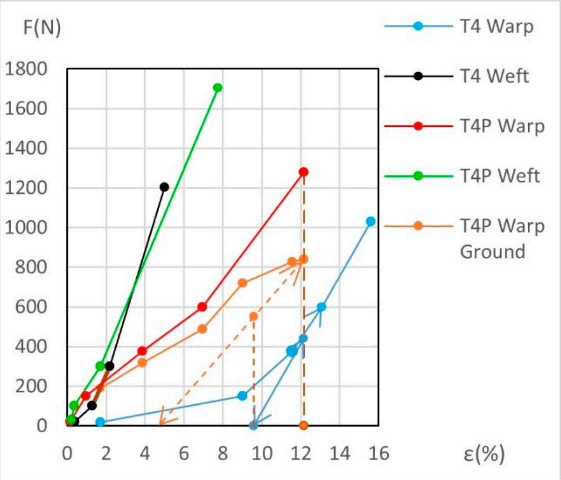

Figure S4. Detailed New T4-T4P Warp. Ground and Canvas Warp with elastic decreasing to F=0.

Detailed evolution of Aged Samples T1P (Polycotton)

At the end of the elastic 3rd phase, the T1 warp (canvas) reaches a force of 651.2 N at an elongation of 20.29%. At this same phase endpoint, the T1P warp reaches 720 N at 18.53% elongation. At 18.53% elongation, the ground layer carries a force of 124.1 N. If the T1P warp begins to contract, the ground also contracts, following the slope of its elastic phase, from 73.2 N to 20 N, and attains the force 0 N at an elongation of ε = 16.62%. At this point (ε = 16.62%), the canvas is still under tension, while the ground is not. If the T1P warp continues to shrink, the canvas follows the same F/ε slope as the T1 warp in its elastic phase, and the force reaches 0 N at an elongation of ε = 2.08%. Between ε = 16.62% and ε = 2.08%, the ground continues to contract and wrinkle, resulting in plastic deformation. See here Figure S5 and in the main article the Figures 8 (left) and 12 (left).

At the end of the elastic 3rd phase, the T1 weft (canvas) reaches a force of 306 N at an elongation of 13.6%. At this same phase endpoint, the T1P weft reaches 496.6 N at 13.6% elongation. At 13.6% elongation, the ground layer carries a force of 190.6 N. If the T1P weft begins to contract, the ground also contracts, following the slope of its elastic phase, from 51.1 N to 21 N, and attains the force 0 N at an elongation of ε = 9.07%. At this point (ε = 9.07%), the canvas is still under tension, while the ground is not. If the T1P weft continues to shrink, the canvas follows the same F/ε slope as the T1 weft in its elastic phase, and the force reaches 0 N at an elongation of ε = 2.03%. Between ε = 9.07% and ε = 2.03%, the ground continues to contract and wrinkle, resulting in plastic deformation. See here Figure S5, and in the main article the Figures 8 (left) and 12 (left).

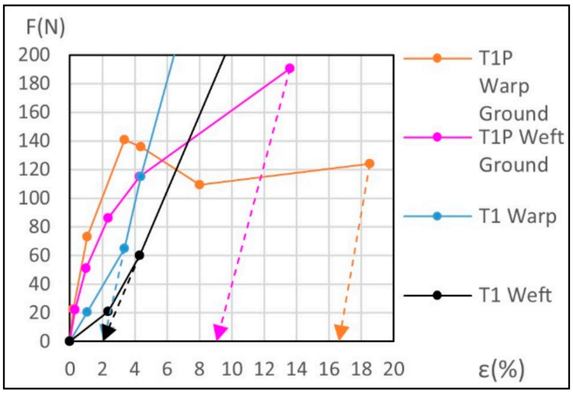

Figure S5. Detailed Aged T1P Ground and T1 Canvas.

Detailed evolution of Aged Samples T2P (Polycotton)

At the end of the elastic 3rd phase, the T2 warp (canvas) reaches a force of 347.2 N at an elongation of 9.42%. At this same phase endpoint, the T2P warp reaches 320 N at 8.08% elongation. At 8.08% elongation, the ground layer carries a force of 32.3 N. If the T2P warp begins to contract, the ground also contracts, following the slope of its elastic phase, from 97.2 N to 20 N, and attains the force 0 N at an elongation of ε = 7.46%. At this point (ε = 7.46%), the canvas is still under tension, while the ground is not. If the T2P warp continues to shrink, the canvas follows the same F/ε slope as the T2 warp in its elastic phase, and the force reaches 0 N at an elongation of ε = 1.78%. Between ε = 7.46% and ε = 1.78%, the ground continues to contract and wrinkle, resulting in plastic deformation. See here Figure S6, and in the main article the Figures 8 (right) and 12 (right).

At the end of the elastic 3rd phase, the T2 weft (canvas) reaches a force of 322 N at an elongation of 13.44%. At this same phase endpoint, the T2P weft reaches 330.8 N at 9.3% elongation. At 9.3% elongation, the ground layer carries a force of 163.7 N. If the T2P weft begins to contract, the ground also contracts, following the slope of its elastic phase, from 94 N to 21 N, and attains the force 0 N at an elongation of ε = 7.33%. At this point (ε = 7.33%), the canvas is still under tension, while the ground is not. If the T2P weft continues

to shrink, the canvas follows the same F/ε slope as the T2 weft in its elastic phase, and the force reaches 0 N at an elongation of ε = 4.84%. Between ε = 7.33% and ε = 4.84%, the ground continues to contract and wrinkle, resulting in plastic deformation. See here Figure S6, and in the main article the Figures 8 (right) and 12 (right).

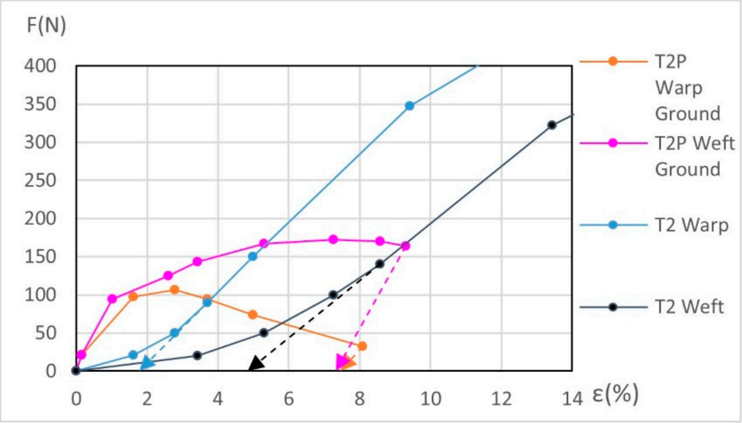

Figure S6. Detailed Aged T2P Ground and T2 Canvas.
